# Leaf rust (*Puccinia recondita* f. sp. *secalis*) triggers substantial changes in rye (*Secale cereale* L.) at the transcriptome and metabolome levels

**DOI:** 10.1186/s12870-024-04726-0

**Published:** 2024-02-13

**Authors:** T. Krępski, A. Piasecka, M. Święcicka, M. Kańczurzewska, A. Sawikowska, M. Dmochowska-Boguta, M. Rakoczy-Trojanowska, M. Matuszkiewicz

**Affiliations:** 1 Department of Plant Genetics, Breeding and Biotechnology, Institute of Biology, Warsaw University of Life Sciences, Warsaw, Poland; 2 Institute of Bioorganic Chemistry, Polish Academy of Sciences, Poznań, 61-704 Poland; 3 Institute of Mathematics, Poznan University of Technology, Poznań, 60-965 Poland; 4 Department of Mathematical and Statistical Methods, Poznań University of Life Sciences, Poznań, 60-637 Poland; 5 Institute of Bioorganic Chemistry, Polish Academy of Sciences, Poznań, 61-704 Poland; 6 Plant Breeding and Acclimatization Institute - National Research Institute, Radzikow, Blonie, 05-870 Poland

**Keywords:** Biotic stress, Fungal disease, Plant immune response, RNA-seq, Differentially accumulated metabolites

## Abstract

**Background:**

Rye (*Secale cereale* L.) is a cereal crop highly tolerant to environmental stresses, including abiotic and biotic stresses (e.g., fungal diseases). Among these fungal diseases, leaf rust (LR) is a major threat to rye production. Despite extensive research, the genetic basis of the rye immune response to LR remains unclear.

**Results:**

An RNA-seq analysis was conducted to examine the immune response of three unrelated rye inbred lines (D33, D39, and L318) infected with compatible and incompatible *Puccinia recondita* f. sp. *secalis* (*Prs*) isolates. In total, 877 unique differentially expressed genes (DEGs) were identified at 20 and 36 h post-treatment (hpt). Most of the DEGs were up-regulated. Two lines (D39 and L318) had more up-regulated genes than down-regulated genes, whereas the opposite trend was observed for line D33. The functional classification of the DEGs helped identify the largest gene groups regulated by LR. Notably, these groups included several DEGs encoding cytochrome P450, receptor-like kinases, methylesterases, pathogenesis-related protein-1, xyloglucan endotransglucosylases/hydrolases, and peroxidases.

The metabolomic response was highly conserved among the genotypes, with line D33 displaying the most genotype-specific changes in secondary metabolites. The effect of pathogen compatibility on metabolomic changes was less than the effects of the time-points and genotypes. Accordingly, the secondary metabolome of rye is altered by the recognition of the pathogen rather than by a successful infection. The results of the enrichment analysis of the DEGs and differentially accumulated metabolites (DAMs) reflected the involvement of phenylpropanoid and diterpenoid biosynthesis as well as thiamine metabolism in the rye immune response.

**Conclusion:**

Our work provides novel insights into the genetic and metabolic responses of rye to LR. Numerous immune response-related DEGs and DAMs were identified, thereby clarifying the mechanisms underlying the rye response to compatible and incompatible *Prs* isolates during the early stages of LR development. The integration of transcriptomic and metabolomic analyses elucidated the contributions of phenylpropanoid biosynthesis and flavonoid pathways to the rye immune response to *Prs*. This combined analysis of omics data provides valuable insights relevant for future research conducted to enhance rye resistance to LR.

**Supplementary Information:**

The online version contains supplementary material available at 10.1186/s12870-024-04726-0.

## Background

 Rye (*Secale cereale* L.) is considered to be the cereal crop most tolerant to abiotic and biotic stresses, including fungal diseases [1]. Among the rye diseases caused by fungi, leaf rust (LR) also known as brown rust, which is an airborne disease caused by the obligate biotrophic basidiomycete *Puccinia recondita* f. sp. *secalis* (*Prs*) (Roberge ex Desmaz), is responsible for significant yield and economic losses [2]. The genetic basis of the resistance to this disease remains relatively unknown and is a major interest of breeders. To date, 16 dominant *Pr* genes (*Pr1-5*, *Pr-d–f*, *Pr-i–l*, *Pr-n*, *Pr-p*, *Pr-r*, and *Pr-t*) on five of the seven rye chromosomes (1R, 2R, 4R, 6R, and 7R) have been identified using Mendelian-based methods [2–5]. The release of rye reference genome sequences (Lo7 and Weining) [6, 7] has allowed researchers to conduct precise analyses at the molecular level. For example, Vendelbo et al. [8, 9] performed a genome-wide association study and mapped five LR resistance-associated quantitative trait loci (QTLs) on chromosome arms 1RS, 1RL, 2RL, 5RL, and 7RS; the two QTLs on chromosome arms 1RS and 7RS were especially important for LR resistance. The main resistance-associated marker on chromosome arm 1RS was physically co-localized with molecular markers delimiting the previously characterized *Pr3* gene. The second region on 7RS contained several nucleotide-binding leucine-rich repeat (NLR)-encoding genes, one of which (provisionally designated as *Pr6*) was similar (at the protein level) to the wheat LR resistance gene *Lr1*, which is on chromosome arm 5DL. However, these results have not been supported by an analysis at the transcriptome level.

In addition to typical resistance genes, the genes controlling benzoxazinoid (BX) biosynthesis (Table S1) are also affected by *Prs* infections [10]. For example, the expression level of *ScBx4*, which encodes a cytochrome P450 monooxygenase, reportedly increases in infected plants at 8, 17, 24, and 48 h post-treatment (hpt). This is in accordance with an earlier finding that a single nucleotide polymorphism (ScBx4_1583) in *ScBx4* is stably associated with the field resistance of adult plants to LR [11]. Transcriptome sequencing (RNA-seq) is a powerful experimental technique for exploring global changes in gene expression in response to various stimuli (e.g., developmental changes and responses to abiotic and biotic stresses) [12]. It has been widely used for studying plant host–pathogen interactions and various diseases, including LR [13–15] in wheat, which is a close relative of rye. Poretti et al. [14] identified 753 genes with expression levels that were uniquely down-regulated in the susceptible isogenic line Thatcher following an infection with LR and powdery mildew. An enrichment analysis of these genes indicated that six major biochemical pathways (nuclear transport, alternative splicing, DNA damage response, ubiquitin-mediated proteolysis, phosphoinositol signaling, and photosynthesis) were suppressed by the diseases. Therefore, the authors concluded that both pathogens can overcome plant immune responses by repressing programmed cell death and responses to cellular damage.

Ji et al. [15] identified 1,455 differentially expressed genes (DEGs) in the wheat–*Agropyron cristatum* 2P addition line II-9-3 infected with LR; most of these DEGs were wheat genes. The Kyoto Encyclopedia of Genes and Genomes (KEGG) analysis and gene set enrichment analysis (GSEA) assigned the DEGs to several pathways, including the following: plant–pathogen interaction, MAPK signaling pathway–plant, plant hormone signal transduction, glutathione metabolism, and phenylpropanoid biosynthesis. Among the *A. cristatum* DEGs, there were many defense-related genes, including genes encoding NLRs, receptor kinases, and transcription factors.

To date, the RNA-seq method has been used to identify genes associated with rye responses to fungal diseases. In 2020, Mahmood et al. [16] conducted an RNA-seq analysis to identify rye DEGs linked to an ergot infection caused by *Claviceps purpurea*. By performing a Gene Ontology (GO) enrichment analysis, the authors detected 228 genes associated with metabolic processes, hydrolase activities, pectinesterase activities, and cell wall modifications. These over-represented groups of genes were considered to be critical for successful parasitism. Tsers et al. [17] detected several genes related to the resistance/susceptibility to *Microdochium nivale*. Their results identified flavonoid-related genes as the most important group of genes mediating the resistance to this pathogen. The susceptibility of plants to *M. nivale* is apparently influenced by the expression of genes encoding lipases and proteins associated with lipase activities. For LR, only one RNA-seq analysis identified rye orthologs of wheat *Lr* genes [18]. The authors determined that *ScLr1_3* and *ScLr1_4* (on chromosome 7R) as well as *ScLr1_8* and *ScRga2_6* (on chromosome 6R) are differentially expressed in three unrelated rye inbred lines infected with LR. Unfortunately, it is unknown whether these genes are the counterparts to *Pr2* and *Pr6*, which are also on chromosome 7R. Apart from these four genes, no other *Lr* genes, including those identified by Vendelbo et al. [8, 9], were differentially expressed. Peng and Yang [19] stated that in wheat infected with LR some NLR are known to be extremely weakly expressed.

In addition to alterations at the transcriptome level, plant immune responses also involve the synthesis of immunity-related metabolites [20]. Phenylpropanoids and their downstream metabolites (flavonoids) are scavengers of reactive oxygen species (ROS) generated in response to environmental stresses [20, 21]. Moreover, several phenolics and the glycosidic forms of flavonols are inhibitors of fungal growth in cereals [22, 23]. There has recently been considerable interest in the antifungal properties of indole-derived BXs [10]. The accumulation of BXs has been correlated with the resistance to various diseases of grasses, including LR in rye [10], head blight caused by *Fusarium* ssp. [24], and corn leaf blight [25]. Nevertheless, the genes and end-products in the BX pathway respond inconsistently to pathogens, indicative of a complex system regulating BX biosynthesis. Accordingly, the relationship between BXs and plant defenses will need to be clarified. The largest and most diverse group of cereal immunity-related metabolites are terpenoids [26]. The thoroughly characterized diterpenes involved in rice–pathogen interactions are momilactones and oryzalexins [27, 28]. Moreover, biogenic amines and their phenol-containing conjugates accumulate rapidly during the interaction between pathogens and plants, including rye [29], barley [30], and wheat [21].

An earlier analysis of the rye metabolome profile led to the identification of groups of defensive metabolites responsive to a nematode attack [31]. A metabolomic analysis was also performed to compare resistant and susceptible wheat genotypes infected with LR [32], thereby revealing metabolite functions potentially related to wheat stripe rust resistance [33].

The objective of this study was to identify and characterize DEGs and differentially accumulated metabolites (DAMs) in three unrelated rye inbred lines infected with compatible and incompatible isolates of *Prs* using several analytical methods, including RNA-seq, quantitative reverse transcription PCR (RT-qPCR), and liquid chromatography and mass spectrometry (LC-MS)-based untargeted metabolomics combined with dedicated bioinformatics approaches. We hypothesize that compatible and incompatible plant-pathogen interactions induce specific changes at the transcriptome and metabolome levels.

## Methods

### Plant materials and inoculation procedure

The following three unrelated rye inbred lines were included in this study: D33 and D39 (both bred by Danko Plant Breeding Ltd., Poland) and L318 (bred in our department). These lines were selected according to their BX contents in early spring (in the developmental stage GS 20–24 according to the Zadoks Cereal Growth Stage, about two weeks after the start of the vegetation) and disease rating performed at maximum brown rust epidemic intensity (end of May), under field conditions (Table S1). Ten germinating seeds (2 days at 22 °C) were added to a sterilized peat substrate in plastic pots, which were then incubated for 10 days in a growth chamber set at 22 °C with a 16-h light (60 µmol m^−2^ s^−1^)/8-h dark cycle. For each rye line, a compatible (CP) and incompatible (ICP) *Prs* strain was selected after preliminary screening 15 single-spore isolates (Table S2). The strains were selected on the basis of detached-leaf inoculations [10] followed by an *in planta* confirmation. The infection types were described by Murphy’s scale, which utilize a 0–4 scale where 0 corresponds to “immune” (no visible reaction); 1 corresponds to “resistant” (minute uredinia surrounded by chlorosis or necrosis); 2 corresponds to “moderately resistant” (small pustules surrounded by chlorosis); 3 corresponds to “moderately susceptible” (moderately large pustules surrounded by chlorosis); and 4 corresponds to “susceptible” (moderately large to large pustules with little or no chlorosis). Prior to inoculating 12-day-old rye plants, each selected *Prs* isolate was resuspended in Novec 7100 engineered fluid (1 mg/ml) in a glass diffuser (Roth, Basel, Switzerland). The control (mock) plants were inoculated with Novec 7100 engineered fluid, but they were otherwise treated the same as the *Prs*-inoculated plants. Immediately after the inoculation, the plants were covered with black boxes to maintain dark and humid (100%) conditions during the 24-h incubation at 18 °C. The plants were then transferred to a growth chamber. The experiment was completed using three biological replicates comprising five plants from one pot. Plant tissue was collected at 20 and 36 hpt, immediately frozen in liquid nitrogen, and stored at − 80 °C. The time points we selected were the same as before [18]. We decided for these time points because at the 20th hpt only haustorium mother cells were observed at the infection site, while at the 36th hpt an additional micronecrotic reaction was observed, indicating an active plant response. Pathogens secrete effectors from specialized feeding structures – haustoria that affect the expression of many genes related to the immune response against fungal pathogens [34, 35].

### Calcofluor white staining to visualize the plant–pathogen interaction

The rye lines were inoculated with the selected *Prs* isolates as described above. Leaf samples were collected at 20, 36, and 72 hpt, fixed, and stained with calcofluor white [10]. The stained leaf materials were examined using the Diaphot fluorescence microscope (Nikon) to detect germinating spores, appressoria, haustorium mother cells (HMCs), and micronecrosis. The infection sites were defined as the sites with an appressorium as well as HMC and/or micronecrosis. Sites containing only an appressorium were not considered. Observations were made for an average of 60 infection sites per leaf sample, usually in three to four replicates (plants). The following three profiles were used to describe plant–pathogen interactions: i, appressorium + HMC; ii, appressorium + HMC + micronecrosis; and iii, appressorium + micronecrosis. Profiles were expressed as percentages.

### RNA isolation

Total RNA was extracted from approximately 100 mg frozen leaves of the mock- and *Prs*-inoculated rye lines (D33, D39, and L318) using the mirVana miRNA Isolation Kit and the Plant RNA Isolation Aid (Thermo Fisher Scientific, Waltham, MA, USA) for the RNA-seq analysis. The GeneMATRIX Universal RNA Purification kit (EURx, Gdańsk, Poland) was used to isolate total RNA for the RT-qPCR analysis. The RNA concentration and purity were estimated using the NanoDrop 2000 spectrophotometer and the Qubit® 2.0 fluorometer (Invitrogen, Waltham, MA, USA).

### RNA-seq analysis

The extracted RNA was sent to Genomed S.A. (Warsaw, Poland) for the RNA-seq analysis, sequence assembly, and primary gene expression analysis. The RNA-seq libraries were prepared using the NEBNext Ultra II Directional RNA Library Prep Kit for Illumina kit (NEB, Ipswich, MA, USA). A total of 54 cDNA libraries.

[3 lines × 3 treatments (CP *Prs*, ICP *Prs*, and mock) × 2 time-points (20 and 36 hpt) × 3 biological replicates] were prepared for the RNA-seq analysis, which was completed using the NovaSeq 6000 system (Illumina) in the PE150 mode. The sequencing reads were filtered using the Cutadapt (version 3.0) program [36] and then quality reports were generated using the FASTQC (version 0.11.8) software [37]. The reads were mapped using the HISAT2 (version 2.2.0) program [38]. As required by HISAT2, short reads (˂20 bp) were removed. Next, the reads were mapped to the *S. cereale* Lo7 reference genome [6]. The following option of the HISAT2 program was applied: library preparation --rna-strandness RF. The number of read pairs mapped to individual genes was determined using the HTseq program [39], with differentiation due to the transcript strand (--stranded = reverse). The genes were annotated on the basis of the gene description file (gff3 file) for the *S. cereale* Lo7 genome [6]. The results were statistically analyzed – detailed information is provided in the section “statistical analysis”. The raw RNA-seq (fastq) data were deposited in the NCBI database (BioProject: PRJNA888031). Several genes were selected for the validation of the RNA-seq data via an RT-qPCR analysis performed using a standard protocol as previously described [10] (Fig. S1; Table S3).

### Metabolomic analysis

To extract metabolites, 2.5 µL of pure DMSO (Sigma–Aldrich, Steinheim, Germany) was added to 1 mg leaf samples, after which 0.5 mM camphorsulfonic acid and 0.5 mM lidocaine (Sigma–Aldrich) were added as internal standards in proportion of 1 µL of standard for 200 µL of DMSO. All frozen samples were homogenized using 1.0 mm zirconia beads (BioSpecProducts, Bartlesville, OK, USA) and Precellys Evolution tissue homogenizer (Bertin Corp, Montigny-le-Bretonneux, France), with a cycle of 2 × 30 s at 8,000 rpm. The samples were centrifuged (15,000 rpm at 4 °C) and the supernatants were collected for the LC-MS analysis, which was performed using the UltiMate 3000 RS system (Dionex, ThermoFisher Scientific) linked to the TIMS-TOF mass spectrometer (Bruker Daltonics, Hamburg, Germany). The chromatographic separation was completed using the BEH RP C18 column (2.1 × 150 mm, 1.8 μm particle size) at 30 °C, with a mobile phase flow rate of 0.25 mL/min. The elution was conducted using water containing 0.1% formic acid (Sigma–Aldrich) (solvent A) and acetonitrile (VWR Chemicals, Fontenay-sous-Bois, France) containing 0.01% formic acid (solvent B). The gradient elution was as follows: 0–5 min, 10–30% B; 5–12 min, 30–100% B; 12–15 min, maintained at 100% B; and 15–15.5 min, the system was returned to starting conditions and re-equilibrated for 5 min. The mass spectrometer was calibrated using sodium formate salt clusters as the internal calibrant prior to each analysis. The mass spectrometer was operated using the following settings: ion source voltage, − 4.5 kV or 4.5 kV; nebulization of nitrogen, 2.2 bar (pressure); and gas flow rate, 10 L/min. The ion source temperature was 220 °C. The spectra were scanned in positive and negative ionization fragmentation modes (ddMSMS) at a range of 95–1,000 m/z and a resolution of > 30,000 full width at half maximum (FWHM). Data were acquired using the Compass HyStar (version 6.0) software (Bruker Daltonic). The raw LC-MS data were processed using MS-DIAL (version 4.74) [40]. The processing steps included peak detection, annotation according to spectral MSMS public metabolomic libraries (http://prime.psc.riken.jp/compms/msdial/main.html), adduct elimination, alignment, and gap filling by compulsion. The raw data from the positive and negative ionization modes transformed to the universal mzXML format are available online (https://box.pionier.net.pl/d/4e8c093c332b41b2ab6e/).

### Statistical analysis

The DEGs and DAMs in each rye line were detected on the basis of the following four comparisons: CP vs. mock and ICP vs. mock at two time-points (20 and 36 hpt) (Table 1).

**Table 1:** Comparisons for the transcriptome and metabolome analyses

Line	Comparison description	Comparison name
D33	CP *vs* mock, 20 hpt	D33_CP_20
	CP *vs* mock, 36 hpt	D33_CP_36
	ICP *vs* mock, 20 hpt	D33_ICP_20
	ICP *vs* mock, 36 hpt	D33_ICP_36
D39	CP *vs* mock, 20 hpt	D39_CP_20
	CP *vs* mock, 36 hpt	D39_CP_36
	ICP *vs* mock, 20 hpt	D39_ICP_20
	ICP *vs* mock, 36 hpt	D39_ICP_36
L318	CP *vs* mock, 20 hpt	L318_CP_20
	CP *vs* mock, 36 hpt	L318_CP_36
	ICP *vs* mock, 20 hpt	L318_ICP_20
	ICP *vs* mock, 36 hpt	L318_ICP_36

*CP* Compatible *Prs* isolate, *ICP* Incompatible *Prs* isolate, *hpt* hours post treatment

The final RNA-seq results were analyzed in the R environment (version 3.6.3) [41] using DESeq2 (version 1.26.0) [42]. In line with DESeq2 default parameters, the statistical significance of differential expression was tested using a Wald test, and the obtained p-values were corrected for multiple testing using the Benjamini and Hochberg method to calculate false discovery rate (FDR). In all our analysis, genes were considered as differentially expressed (DEGs) if meet the following parameters: |log2(fold-change)| ≥ 2, (FDR) < 0.01, and BaseMean (BM) - the average of the normalized count values ≥ 50. These criteria were selected to avoid false positives. For the KEGG analysis of significant DEGs, the KEGG internal annotation tool BlastKOALA (database: Eukaryotes) was used [43]. The GO enrichment analysis was performed using the software available on the Triticeae-Gene Tribe website [44]. For the analysis, p-values were adjusted according to the Bonferroni-Hochberg correction method and an FDR of 0.05 was applied. The Venn online tool (https://bioinformatics.psb.ugent.be/webtools/Venn/) was used to visualize the relationships between the comparisons of DEGs and DAMs.

The processed metabolomic data were normalized via a log_2_ transformation and missing values were replaced (1/10 of the minimum peak height for all samples). Experimental samples were compared with “blank” samples containing only extraction buffer to eliminate background noise due to the buffer. Curated data tables for the positive and negative ionization modes were combined using MSCleanR and subjected to an ANOVA with FDR correction. The signal intensities were visualized using MetaboAnalyst [45]. For each comparison, the DAMs between the inoculated and control plants were selected according to the following criteria: FDR < 0.01 and |log_2_(fold-change)| > 0.58. The processed LC-MS data are available in Table S4.

### Integration of transcriptomic and metabolomic analyses

Signals corresponding to the DAMs and DEGs were imported into the Joint-Pathway Analysis module in MetaboAnalyst 5.0 to screen for relationships between the DAMs and DEGs. The DAMs were imported into MetaboAnalyst as a peak list profile with fold-change values and p-values for scoring, whereas the DEGs (fold-change values) were imported by Entrez ID. Metabolites over-represented at the pathway level were ranked, with a mass tolerance of 5 ppm. Additionally, the Mummichog 2 algorithm was used, with the p-value cutoff set at 0.05 on the basis of the *Oryza sativa japonica* reference metabolome in the KEGG database [46]. Significantly enriched metabolic pathways were identified using Fisher’s method involving an integrated pathway p-value < 0.1 across multiple comparisons (Table 3) with at least one DAM and one DEG associated with the pathway.

### Correlation network comprising DEGs and DAMs

A combined correlation network was constructed on the basis of three separate correlation networks (for DAMs, DEGs, and both DAMs and DEGs) using the WGCNA package in R [47] and then visualized using Cytoscape [48]. The most highly correlated compounds were grouped according to the first two networks mentioned above, whereas the connections between the compounds were determined on the basis of the third network. To construct each network, the Pearson correlation matrix was transformed into an adjacency matrix using a power function of 9, 8, and 14 for the DAMs, DEGs, and both DAMs and DEGs, respectively, according to the scale-free topology criterion [47]. Modules, which are groups of highly correlated compounds, were detected by clustering using the dynamic tree cut algorithm and the topological overlap matrix (TOM) for the DAMs and DEGs. Thus, the interconnections between nodes were determined on the basis of the correlation network for the DAMs and the correlation network for the DEGs, which included circles for the nodes and the module names [49]. The nodes divided into eight modules are provided in Table S11. All connections between nodes were derived from the network with nodes for both DAMs and DEGs. The connections between compounds as well as their Pearson correlation coefficients and the TOM values are presented in Table S11. Hubs, which were defined as highly connected metabolites and genes, were selected as nodes with the most connections between metabolites or genes according to the network for both DAMs and DEGs. The degree of each node is listed in Table S11. Moreover, intergroup hubs were defined as follows.

A simple graph $G$ consists of a non-empty finite set $V$ of elements called nodes (or vertices) and a finite set $E$ of distinct unordered pairs of distinct elements of $V$ called edges. We call $V$ the node set and $E$ the edge set of $G$. An edge $\{v, w\}$ is said to join the nodes $v$ and $w$. The degree of a node $v$ of $G$ is the number of edges incident with $v$, and is written as $\text{deg}\left(v\right)$ [50].

Let $G({V}_{1}\cup {V}_{2},E)$ denote the correlation network graph with ${V}_{1}\cup {V}_{2}$ nodes and $E$ edges, where ${V}_{1}$ denotes the nodes belonging to one group and ${V}_{2}$ denotes the nodes belonging to the other group. Each of the nodes ${v}_{i,n}\in {V}_{i}$ for $i = 1, 2, n = 1, 2,\dots ,{N}_{i}$, where ${N}_{i}$ is the number of nodes in the $i$th group, belongs to one of the disjoint subgraphs (modules) ${M}_{i,k}$ for$k = 1, 2,\dots ,{K}_{i}$, where ${K}_{i}$ is the number of groups in $i$th group containing only nodes from set${V}_{i}.$

The external degree of node ${v}_{i,n}\in {V}_{i}$ is defined as the number of its incident edges connecting it to the nodes belonging to the other set ${V}_{j}$, where $j \ne i$, and is denoted as$\text{outdeg}\left({v}_{i,n}\right).$

The external degree of module ${M}_{i,k}\in {V}_{i}$ is defined as the number of nodes from set ${V}_{j},$ where $j \ne i$, that are connected by an incident edge to the nodes belonging to module ${M}_{i,k}$. The external degree of the module is defined as $\text{outdeg}\left({M}_{i,k}\right)$.

We call node ${v}_{i,n}\in {M}_{i,k}$ an intergroup hub if and only if

$$\frac{\text{o}\text{u}\text{t}\text{d}\text{e}\text{g}\left({v}_{i,n}\right)}{\text{outdeg} \left({M}_{i,k}\right)}\cdot 100\% \ge 20\%, \text{and}\text{ outdeg}\left({v}_{i,n}\right) \ge 3.$$

Without a loss of generality, the definition can be generalized to N groups (e.g., into three groups by adding proteins to metabolites and genes). All analyses were performed using our in-house scripts in R.

## Results

### *Puccinia recondita* profiles on rye inbred lines inoculated with compatible and incompatible isolates

**Fig. 1:**
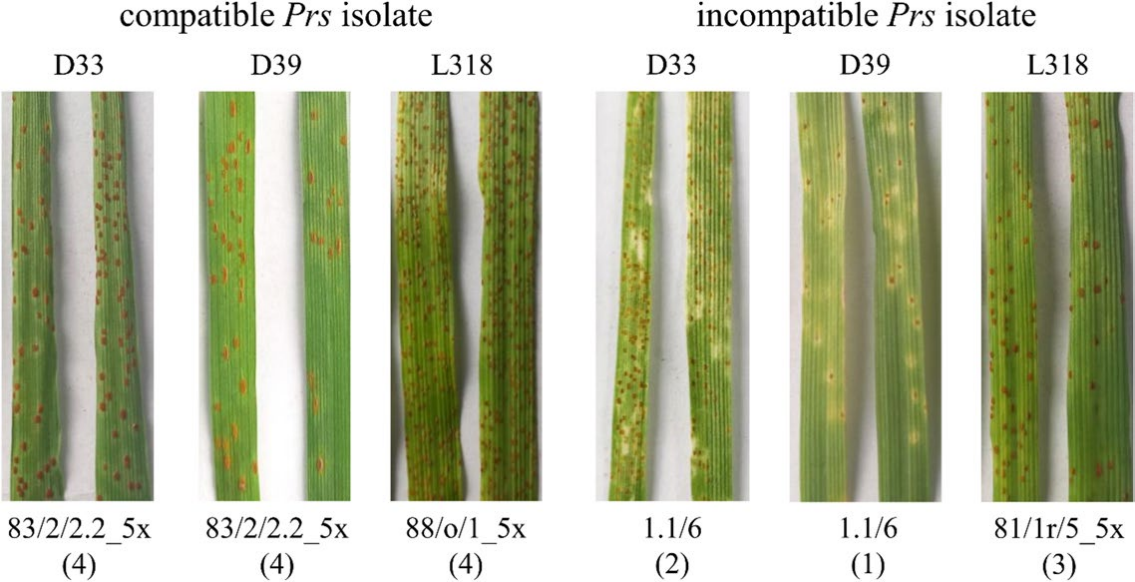
Types of interactions between rye inbred lines and *Prs* isolates. Macroscopic examination of LR symptoms at 10 days after the inoculation of rye inbred lines, D33, D39 and L318, with compatible and incompatible *Prs* isolates. The infection types determined using the following 0–4 scale [51] are provided in parentheses: 0 = immune (no visible reaction); 1 = resistant (minute uredinia surrounded by chlorosis or necrosis); 2 = moderately resistant (small pustules surrounded by chlorosis); 3^*^ = moderately susceptible (moderately large pustules surrounded by chlorosis); and 4 = susceptible (moderately large to large pustules with little or no chlorosis); ^*)^ for line L318, an infection type “3” was treated as a partially incompatible reaction

Three rye inbred lines were infected with the following four isolates derived from single spores: 83/2/2.2_5x (compatible), 1.1/6 (incompatible for lines D33 and D39), 88/o/1_5x (compatible for line L318), and 81/r/5_5x (partially incompatible for line L318, infection type – “3”) (Fig. 1). Of all the isolates tested so far, isolate 81/r/5_5x induced the highest resistance of line L318 to *Prs*.

**Fig. 2:**
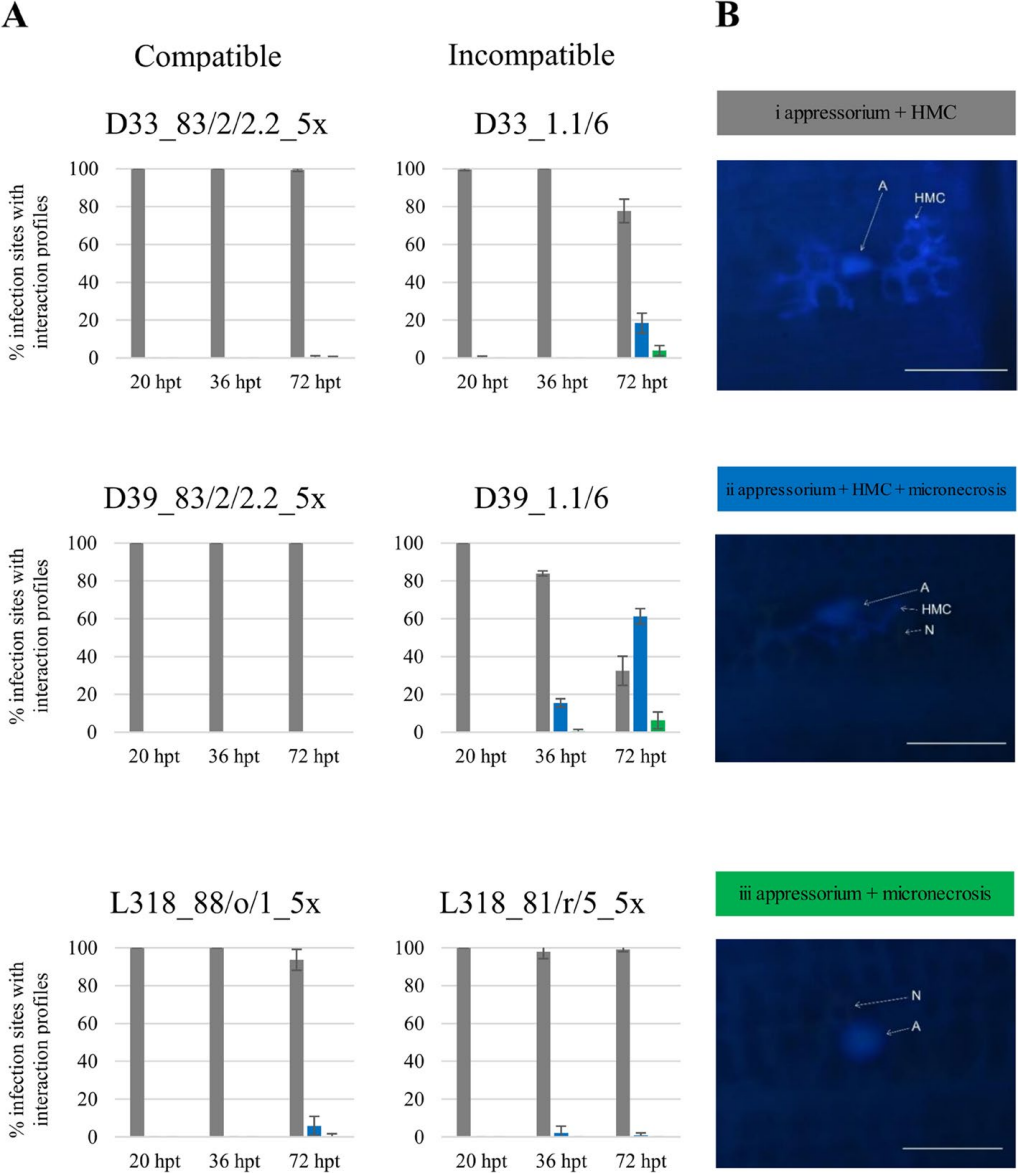
Compatible and incompatible interactions between three rye inbred lines (D33, D39, and L318) and *Prs.* **A** Plant–pathogen interaction profiles for the seedlings of rye inbred lines D33, D39, and L318 inoculated with compatible and incompatible *Prs* isolates. The results show the average percentage of infection sites with profiles i – iii, with standard deviation. Observations were made for average of 60 infection sites per leaf sample (minimum 18, maximum 200) in three – four replicates. Bar colours: grey – profile i (appressorium + HMC), blue – profile ii (appressorium + HMC + micronecrosis) and green -  profile iii (appressorium + micronecrosis). **B** Example of the profiles at 72 hpt in line D39 inoculated with isolate 1.1/6; samples were stained with calcofluor white and examined using a fluorescence microscope. A: appressorium; HMC: haustorium mother cell; N: micronecrosis. Bar = 100 μm

Plant–pathogen interactions were analyzed at 20, 36, and 72 hpt. At 20 hpt, no differences in plant-pathogen interaction were observed between lines or between compatible and incompatible reactions (Fig. 2). At 36 hpt, differences between compatible and incompatible reactions were observed in line D39, these were the first micronecrosis in the incompatible reaction (profile ii: compatible − 0%, incompatible − 15.5%). At 72 hpt, very large differences between compatible and incompatible reactions were observed in line D39 (profile ii: compatible − 0%, incompatible − 61.3%; and profile iii: compatible − 0%, incompatible − 6.2%) and large differences in line D33 (profile ii: compatible − 0.4%, incompatible − 18.4%; profile iii: compatible − 0.3%, incompatible − 3.8%). In line L318, the differences between compatible and incompatible responses were very small. Our analysis was supported by the susceptibility profiles determined at 10 days post-inoculation (Fig. 1).

Considering these results, we examined the changes at the molecular level during the infection of rye inbred lines D33, D39, and L318 by compatible and incompatible *Prs* isolates to clarify the mechanisms underlying the immune responses of the susceptible and resistant rye genotypes infected with *Prs*.

### Transcriptomic analysis of rye infected with leaf rust

We sequenced 54 libraries and generated more than 2,642 million raw 150-bp paired-end reads (approximately 48 million reads per sample). After trimming reads and filtering for quality, we obtained 2,639 million high-quality paired-end clean reads (average of 48 million reads per sample). The average GC content was 53.2%. Approximately 90.6% of the high-quality reads were mapped to the Lo7 rye reference genome [6]. Of these mapped reads, approximately 88% were uniquely mapped to a single locus. The data were processed appropriately for an RNA-seq analysis. A transcriptomic approach was used to investigate the differences in the responses of the three rye inbred lines to compatible and incompatible *Prs* isolates. We selected two time-points because they corresponded to different *Prs* developmental stages (Fig. 2). In total, 877 unique differentially expressed genes (DEGs) were identified at 20 and 36 h post-treatment. Of 877 unique DEGs, 562 (64%) were present only once in only one of 12 comparisons and the remaining 315 (36%) - appeared in more than one comparison, usually in two or three comparisons; maximum six in case of gene *SECCE7Rv1G0460350* encoding ammonium transporter. As a result, the total number of transcripts was 1255 (Table S5, S7).

**Fig. 3:**
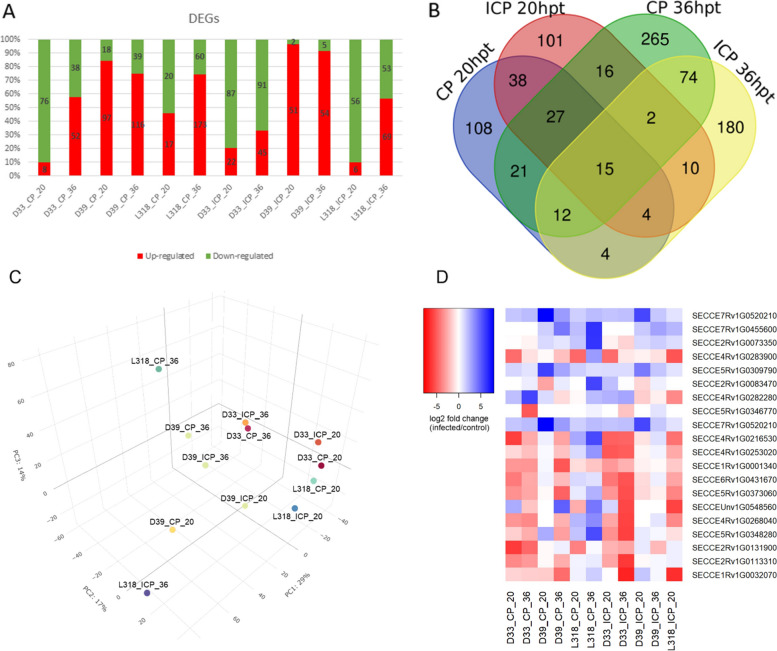
Number of DEGs after infections with CP and ICP *Prs* isolates (20 and 36 hpt). **A** Total number of DEGs (1255) responsive to *Prs*. **B** Venn diagram presenting the DEGs grouped according to the changes in their expression relative to the type of response and time-point. **C** Similarities in the transcriptomic responses to both CP and ICP *Prs* isolates among the three rye genotypes and the two time-points. **D** Heatmap representing changes in the expression of the most important DEGs

The plant–pathogen profiles were similar among the analyzed rye lines. Additionally, approximately 100 DEGs were detected in the compatible and incompatible interactions. The L318_CP_20 comparison had the fewest DEGs (37), whereas the L318_ICP_20 comparison had the most DEGs (233) (Fig. 3A; Table S6). These findings (i.e., relatively few DEGs) were due to the very restrictive filtering parameters that were applied, which allowed us to identify important genes affected by LR in response to the compatible and incompatible *Prs* isolates. For the comparisons involving lines D39, up-regulated genes were the predominant DEGs (especially in the D39_ICP_20 and D39_ICP_36 comparisons), while only in half of the comparisons involving line L318 up-regulated DEGs dominated. Conversely, for the comparisons involving line D33, down-regulated genes were the main DEGs (especially in the D33_CP_20 and D33_CP_36 comparisons) (Fig. 3A). These differences suggest the observed changes may depend on the genetic background of the lines and the type of reaction (Fig. 3C).

### Molecular signature of the rye response to compatible and incompatible *Prs* isolates

To clarify the complexity of rye susceptibility and resistance to LR and reveal the differences between the reaction types, we analyzed gene regulatory networks associated with the rye response to compatible and incompatible *Prs* isolates. Among the identified DEGs, there were more genes affected by CP *Prs* than genes affected by ICP *Prs* (394 vs. 291 genes, with 192 overlapping genes). The proportions were similar for the up-regulated genes after the infection with CP *Prs* (257 vs. 105 genes, with 114 overlapping genes). However, the analysis of the down-regulated genes indicated that the infection with CP *Prs* decreased the expression of fewer genes than the infection with ICP *Prs* (169 vs. 211, with 64 overlapping genes; Table S7).

The analysis of the expression dynamics following the *Prs* infection revealed the changes in expression [i.e., log_2_(fold-change)] ranged from 7.86 to − 6.80 (Fig. 3D; Table S8). The most strongly up-regulated gene was that encoding a kaurene synthase (*SECCE7Rv1G0520210*) found in D39 line during compatible interaction at 20 hpt. The most strongly down-regulated gene encoded an expansin protein family member (*SECCE1Rv1G0032070*) and was detected in line D33 during the incompatible interaction at 36 hpt. The up-regulated genes were major fraction of DEGs in lines D39 and L318 infected with CP *Prs*. Two of these genes (both in line L318), namely *SECCE2Rv1G0073350* and *SECCE4Rv1G0283900*, encoded a peroxidase (PO) and acid invertase 1, respectively. In addition to their high log_2_(fold-change) values, they also had a high BM value (> 600). In contrast, the down-regulated genes were the dominant DEGs in line D33 infected with ICP *Prs*. In this group, the genes with the highest log_2_(fold-change) values had relatively low BM values (from 50 to 3022; BM mean is 377 and median - 141).

The investigation of the genetic factors underlying the compatible and incompatible interactions with *Prs* revealed intriguing expression patterns for specific genes. Our analysis identified seven common genes among the three inbred rye lines that were associated with compatible interactions at all time-points. Within this group, the expression levels of the following three genes were consistently up-regulated in all lines: *SECCE7Rv1G0520220* (glycosyltransferase), *SECCE6Rv1G0429310* (beta-1,3-glucanase), and *SECCE7Rv1G0520230* (cytochrome P450). Conversely, only *SECCE7Rv1G0457810* (thiopurine S-methyltransferase) had a down-regulated expression level in all lines. Interestingly, there were no common gene groups for the incompatible interactions among the analyzed rye lines (Table S5; S9). Several genes common to all lines (Table S9) were selected for the RT-qPCR analysis performed to validate our RNA-seq data. The RT-qPCR data were highly consistent with the RNA-seq data (Fig. S1).

In addition to the DEGs that were common to all three rye inbred lines at every time point, we identified four genes (*SECCE1Rv1G0039520*, *SECCE4Rv1G0273590*, *SECCE5Rv1G0367900*, and *SECCE7Rv1G0491620*) coding for the same type of protein, specifically the NAC domain-containing protein. These genes were specific to CP response. Similarly, five genes (*SECCE1Rv1G0057050*, *SECCE4Rv1G0232880*, *SECCE7Rv1G0507990*, *SECCE7Rv1G0508030*, and *SECCE7Rv1G0508100*) encoding xyloglucan endotransglucosylase/hydrolases (XTH), which are typically associated with ICP, were differentially regulated in all three rye inbred lines. A number of genes were exclusively found in the D33 line, including ten genes (*SECCE1Rv1G0043580, SECCE1Rv1G0052820, SECCE1Rv1G0058340, SECCE3Rv1G0200810, SECCE4Rv1G0250540, SECCE5Rv1G0361700, SECCE6Rv1G0378580, SECCE6Rv1G0378610, SECCE6Rv1G0378630* and *SECCEUnv1G0564610*) encoding chlorophyll *a*/*b*-binding proteins (CabBP), and four genes (*SECCE1Rv1G0027760, SECCE2Rv1G0116400, SECCE7Rv1G0454360* and *SECCE7Rv1G0496900*) encoding Aquaporin and Aquaporin-like proteins, which showed down-regulation in the CP and ICP interactions, respectively (Table S5).

### Time-point-specific DEGs related to the rye responses to compatible and incompatible *Prs* isolates

Time-point-specific DEGs related to the rye responses to compatible and incompatible *Prs* isolates.

Our goal was to identify time-point-specific transcriptomic changes caused by the compatible and incompatible *Prs* isolates. A total of 229 and 432 unique DEGs were detected for the compatible interaction at 20 and 36 hpt, respectively. Similarly, 213 and 301 unique DEGs were detected for the incompatible interaction at 20 and 36 hpt, respectively (Table S5). One intriguing group consisted of genes associated with a particular response to LR (Fig. 3B). Specifically, for the compatible interaction, we identified 108 and 265 LR response-related DEGs at 20 and 36 hpt, respectively. For the incompatible interaction, there were 101 and 180 LR response-related DEGs at 20 and 36 hpt, respectively. At both time-points of the incompatible interaction, the genes were mostly down-regulated. For the compatible interaction, the DEGs at 20 hpt were mainly down-regulated genes, but at 36 hpt, the DEGs were primarily up-regulated genes. In group 108 (CP_20hpt unique), the significantly enriched GO categories included translation, response to light, and rRNA binding. In contrast, the main enriched GO categories in group 265 (CP_36hpt unique) were glutathione metabolism, salicylic acid (SA) signaling, and jasmonic acid (JA) signaling. Notably, during the incompatible interactions, completely different GO categories were enriched, reflecting the specificity of the rye response to *Prs*. Specifically, after 20 hpt, the GO categories enriched among the genes in group 101 (ICP_20hpt unique) were lipid transport and plant cell wall biogenesis, whereas these categories were not enriched in group 180 (ICP_36hpt unique). Instead, asparagine biosynthetic process and response to JA were slightly enriched GO categories (Table S6).

Our analyses identified several genes (15 DEGs) that were present in all types of comparisons, encompassing both CP and ICP responses as well as both time points. Except for two genes: *SECCE7Rv1G0460350* (coding for ammonium transporter) and *SECCE4Rv1G0248210* (coding for cytochrome P450) which were identified as DEGs in all three rye lines, all the remaining thirteen genes were differentially expressed in one (D39 or L318) or two (usually D39 and L318) lines. This observation suggests that these genes may play a substantial role in developmental processes of the pathogen. The genes in this group encoded proteins involved in cell wall modifications, including WIR1a, endo-1,3-beta-glucanase, and 1-deoxy-D-xylulose 5-phosphate synthase (DXS), as well as genes belonging to the CYP450 family involved in NADPH- and/or O_2_-dependent hydroxylation reactions. Interestingly, all of the genes common to the CP and ICP interactions were up-regulated DEGs (Table 2; Table S5).

**Table 2:** Common pool of genes affected by LR identified in both interactions (CP and ICP) and both time points (20 hpt and 36 hpt)

No	Gene ID	Encoded protein	Differentially expressed in comparison:
1	SECCE7Rv1G0458590	Ice recrystallization inhibition protein-like protein	D33_ICP_20; D33_CP_20 D33_CP_36; D39_CP_36 D39_ICP_36
2	SECCE3Rv1G0160690	basic helix-loop-helix (bHLH) DNA-binding superfamily	D33_ICP_36; D33_CP_36 D39_ICP_20; D39_CP_20
3	SECCE7Rv1G0460350	Ammonium transporter	D39_ICP_20; D39_CP_20 D39_ICP_36; D39_CP_36 L318_ICP_36; L318_CP_36
4	SECCE2Rv1G0142080	1-deoxy-D-xylulose 5-phosphate synthase	D33_ICP_20; D39_ICP_20 D39_CP_20; D39_ICP_36 D39_CP_36
5	SECCE5Rv1G0322060	Indole-3-glycerol phosphate synthase	D39_ICP_20; D39_CP_20 D39_ICP_36; D39_CP_36
6	SECCE2Rv1G0117990	Cysteine protease	L318_ICP_20; L318_CP_20 L318_ICP_36; L318_CP_36
7	SECCE7Rv1G0460090	Aldo/keto reductase family protein	D39_ICP_20; D39_CP_20 D39_ICP_36; D39_CP_36
8	SECCE6Rv1G0429650	Endo-1,3-beta-glucanase	D39_ICP_20; D39_CP_20 D39_ICP_36; D39_CP_36 L318_CP_20; L318_CP_36
9	SECCEUnv1G0532270	tRNA-specific 2-thiouridylase MnmA	D39_ICP_20; D39_CP_20 D39_ICP_36; D39_CP_36 L318_CP_36
10	SECCEUnv1G0568520	WIR1a	D39_ICP_20; D39_CP_20 D39_ICP_36; D39_CP_36 L318_CP_36
11	SECCEUnv1G0532290	WIR1a	D39_ICP_20; D39_CP_20 D39_ICP_36; D39_CP_36
12	SECCE5Rv1G0302060	WIR1a	D39_ICP_20; D39_CP_20 D39_ICP_36; D39_CP_36 L318_CP_36
13	SECCE4Rv1G0285880	Cytochrome P450, putative	D39_ICP_20; D39_CP_20 D39_ICP_36; D39_CP_36
14	SECCE7Rv1G0462250	Cytochrome P450	D39_ICP_20; D39_CP_20 D39_ICP_36; D39_CP_36
15	SECCE4Rv1G0248210	Cytochrome P450	D33_ICP_36; D39_ICP_20 D39_CP_20; D39_CP_36 L318_CP_36

### Functional classification of leaf rust-regulated genes

There were many genes with changes in expression due to LR during compatible and incompatible interactions. These genes covered a substantial portion of the genome. To functionally classify the DEGs, BlastKOALA was used and a GO enrichment analysis was performed. The characterization of gene functions allowed us to visualize the regulatory trends in different biological pathways affected by *Prs* development.

First, we analyzed the enrichment of all 877 unique DEGs from all comparisons to determine which processes are crucial for *Prs* development independent of the reaction type. Using BlastKOALA, we assigned 340 of the 877 unique DEGs to 18 KEGG categories. The categories with the most DEGs were “Biosynthesis of other secondary metabolites” (38 DEGs), “Carbohydrate metabolism” (30 DEGs), and “Genetic information processing” (30 DEGs). The categories with the fewest DEGs were “Cellular processes” (three DEGs) and “Nucleotide metabolism” (two DEGs) (Table S5).

**Fig. 4:**
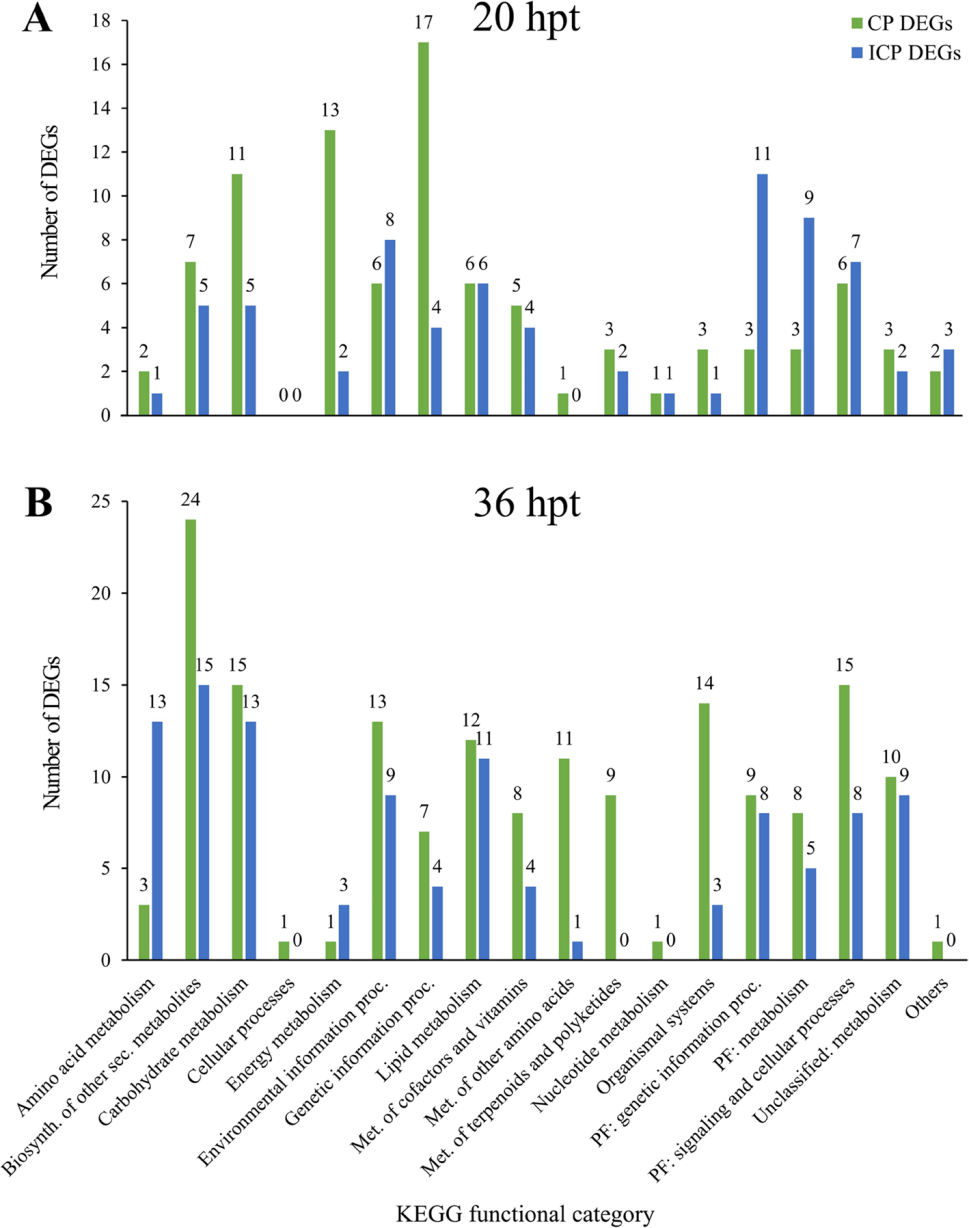
KEGG-based functional classification of DEGs in CP and ICP interaction. DEGs were analyzed separately (**A**) at 20 hpt and (**B**) at 36 hpt by using BlastKOALA method; biosynth. - biosynthesis; met. - metabolism; PF - protein families; proc. - processing; sec. – secondary

 In this study, we wanted to highlight the differences in the enriched KEGG categories among the DEGs in the compatible and incompatible interactions (Fig. 4). The CP DEGs were the dominant DEGs at 20 and 36 hpt and were assigned to most of the KEGG functional categories (10 and 16 of the 18 KEGG categories, respectively). There was an equal number of DEGs for the CP and ICP reactions only at 20 hpt and exclusively in three categories, namely “Cellular processes”, “Lipid metabolism”, and “Nucleotide metabolism”. At 20 hpt, there was a large increase in the number of CP DEGs (2- to 6-times more) in the following three categories: “Carbohydrate metabolism”, “Energy metabolism”, and “Genetic information processing”. For the ICP DEGs at 20 hpt, the KEGG categories with a substantial increase in the number of genes (at least 2-times more) were “Protein families: genetic information processing” and “Protein families: metabolism”. The “Metabolism of other amino acids” category lacked ICP DEGs, but it included one CP DEG (Fig. 4A). At 36 hpt, CP DEGs were over-represented (i.e., > 2-times more abundant than ICP DEGs) in the following categories: “Metabolism of cofactors and vitamins”, “Metabolism of other amino acids” and “Organismal systems”. The ICP DEGs were > 2-times more abundant than the CP DEGs in two categories at 36 hpt, namely “Amino acid metabolism” and “Energy metabolism” but the opposite trend in these categories was observed at 20 hpt. Four categories, including “Metabolism of terpenoids and polyketides” comprised only CP DEGs (Fig. 4B).

### Metabolite profiling following an infection with *Prs*

**Fig. 5:**
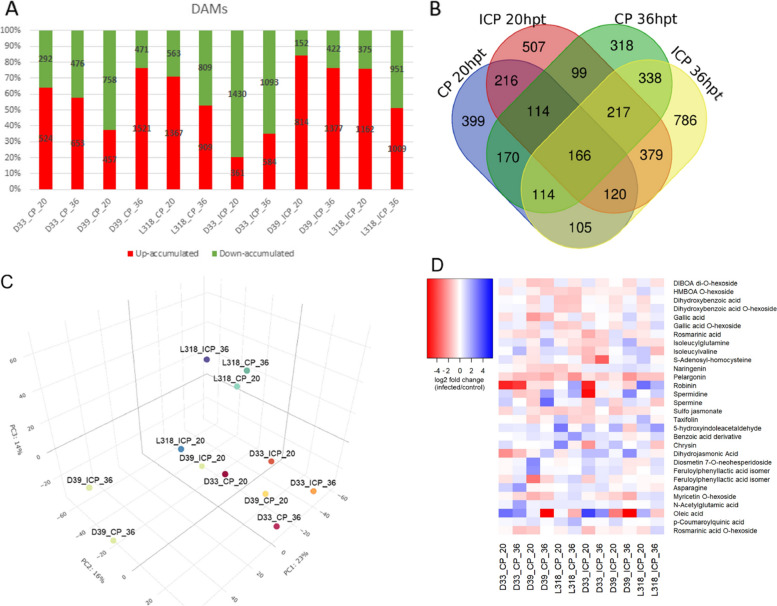
Number and characteristics of DAMs. **A** Number of DAMs. **B** Time-point-specific and common DAMs for the pooled genotypes infected with CP or ICP *Prs*. **C** Similarities in the metabolomic responses to both CP and ICP *Prs* isolates among the three rye inbred lines and at the two time-points. **D** Heatmap representing changes in the expression of the most important DAMs

To support our transcriptomic data that identified many DEGs related to plant metabolism, we completed a comparative metabolomic analysis of rye infected with *Prs*. The three rye inbred lines exhibited diverse metabolomic responses to *Prs*. Line D33 infected with CP *Prs* had the fewest number of DAMs at both time-points, whereas line L318 had the highest number of DAMs. The infection with ICP *Prs* resulted in the significant decrease in proportion of the number of up-accumulated metabolites in D33 in comparison to CP response. The opposite effect was observed in line D39 (i.e., increase in the up-accumulation of metabolites with ICP in comparison to CP treatment) (Fig. 5A).

The metabolomic profiles of L318 after *Prs* infection (i.e., identified DAMs) is distant to profiles of the other two lines. The most diverse immune response at the metabolomic level was detected for D39 at 36 hpt, whereas at 20 hpt, the response of D39 was similar to that of D33 (Fig. 5C).

Because the time-point was revealed to have the largest effect on the groupings among treatments, we determined the number of common and specific DAMs, differentiating between the time-points and reaction types. The metabolomic response was highly treatment-specific. The ICP reaction resulted in the largest number of specific DAMs at 20 and 36 hpt for all pooled genotypes (Fig. 5B). Additionally, there were more common DAMs between the two time-points for the ICP infection (379) than between the two time-points for the CP infection (170). There were considerably more common DAMs (166) than common DEGs (15). These 166 common DAMs related to rye immunity included flavonoids (e.g., catechins and glycosides of kaempferol and quercetin), phenylpropanoids (e.g., sinapic and rosmarinic acids), as well as polymine spermidine and spermine (Fig. 5B and D; Table S12). The amino acids threonine and asparagine were also identified among the common metabolites. Interestingly, sulfo-jasmonate was a common DAM, while JA was a DAM specific to the 20 hpt time-point of the CP infection. Among the 170 DAMs common to both time-points of the CP infection, benzoic acid derivatives (benzoic acid and gallic acid as well as their hexosides) were specific to the CP infection. Both DIBOA diglucoside and HMBOA glucoside were also associated with the CP infection. This set of DAMs also included the flavonoids naringenin and taxifolin. The ICP-specific DAMs (among 379 signals) included the flavonoid chrysin and peptides (e.g., isoleucylglutamine, isoleucylvaline, and S-adenosyl-homocysteine).

### Functional interactions among DAMs and DEGs

To combine the RNA-seq and metabolomic profiling results, the DEGs and DAMs from all comparisons were subjected to a joint-pathway enrichment analysis involving the KEGG pathway database (https://www.genome.jp/kegg/pathway.html) and MetaboAnalyst (Table 3; Table S4). First, the highly enriched pathways identified on the basis of the annotation of only one data type (metabolites or transcripts) were eliminated. Only two pathways (“Phenylpropanoid biosynthesis” and “Diterpenoid biosynthesis”) were commonly enriched among the genotypes. Most of the matched pathways were specific to certain treatments. Phenylpropanoids were the most commonly identified DAMs among treatments. Examples include phenylalanine, tyrosine, hydroxycinnamic acids and their amines and aldehyde derivatives, as well as chlorogenic acids. Monolignols (annotated as syringin and coniferin and their derivatives) were also matched. The PO-encoding DEGs were associated with the metabolites from the “Phenylpropanoid biosynthesis” pathway. “Diterpenoid biosynthesis” was mainly related to “Gibberellin biosynthesis.” The infection with *Prs* induced changes in the accumulation of several metabolites related to “Gibberellin biosynthesis,” but only one or two related transcripts in this pathway were matched.

**Table 3:**
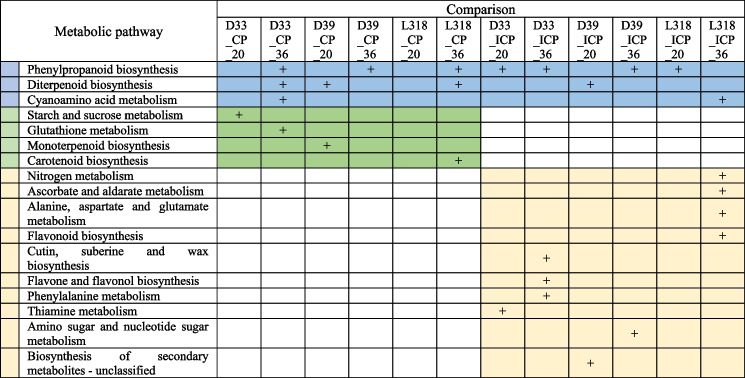
Joint-pathway enrichment analysis^a^ of the DAMs and DEGs

^a^The analysis was performed on the basis of the enriched KEGG metabolic pathways for all 12 comparisons. Only pathways containing both metabolites and transcripts were selected (merged *p*-value < 0.1). The complete functional enrichment results and annotated compounds are provided in Table S10. D33, D39, and L318 refer to rye inbred lines; CP and ICP represent compatible and incompatible isolates, respectively; 20 and 36 refer to the plants harvested at 20 and 36 hpt, respectively. Blue color indicates metabolic pathways enriched for both CP and ICP treatments, green color indicates metabolic pathways enriched for CP treatments and yellow color indicates metabolic pathways enriched for ICP treatments

The interaction between the ICP isolate and lines D33 and L318 at 36 hpt had highly specific enriched pathways. The immune response of D33 to the ICP isolate at 36 hpt was exclusively associated with “Cutin, suberine and wax biosynthesis”, “Flavone and flavonol biosynthesis” and “Phenylalanine metabolism”. Similarly, the interaction between the ICP isolate and line L318 at 36 hpt was reflected by the enrichment of “Nitrogen metabolism”, “Alanine, aspartate and glutamate metabolism”, “Flavonoid biosynthesis” and “Ascorbate and aldarate metabolism”. Furthermore, the “Ascorbate and aldarate metabolism” pathway was associated with the “Ascorbate biosynthesis” module. However, the central metabolite ascorbic acid was not annotated. “Starch and sucrose metabolism” was enriched for the interaction between line D33 and the CP isolate at 20 hpt, while another sugar-related pathway (“Amino sugar and nucleotide sugar metabolism”) was enriched for line D39.

### Integration of the transcriptomic and metabolomic changes in rye in response to *Prs*

**Fig. 6:**
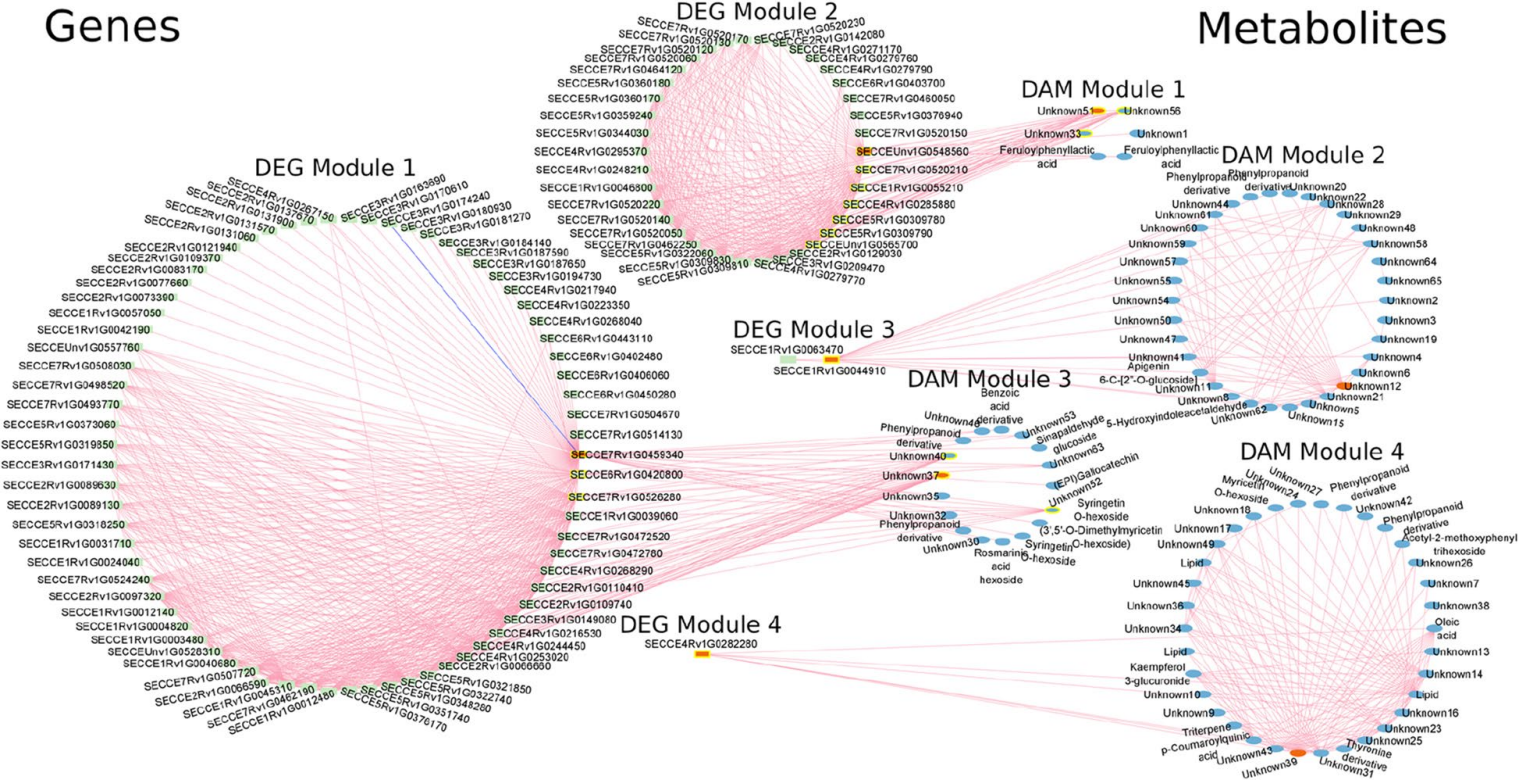
Correlation network of DEGs and DAMs. The genes are represented by green squares with labels, whereas the metabolites are represented by blue ellipses with labels. Hubs are indicated in orange, with intergroup hubs containing a yellow border. Edges link highly correlated compounds. Modules of compounds are indicated by circles. Only edges corresponding to elements of the topological overlap matrix (greater than 0.55) are shown, both within and between modules; pink and blue edges indicate positive and negative correlations, respectively. Only one negative correlation was detected

 To more comprehensively characterize the rye response to *Prs*, a transcript–metabolite correlation network was constructed for all DEGs and DAMs, but only the DEGs and DAMs connected to other features are presented in Fig. 6.

The correlations among the DEGs, DAMs, and their interactions were mostly positive. Only one negative correlation, which was between the dentin sialophosphoprotein-related gene (*SECCE3Rv1G0174240*) and the UGT gene (*SECCE7Rv1G0459340*), was detected in Module 1. The UGT gene (*SECCE7Rv1G0459340*) was both a hub and an intergroup hub of Module 1. The *SECCE6Rv1G0420800* and *SECCE7Rv1G0526280* genes were also intergroup hubs of this module. There were four modules for the correlations among the DAMs and for the correlations among the DEGs. The largest module within the DEGs was Module 1, which included mostly genes responsible for cell wall remodeling. Strong interactions were observed within modules. More specifically, the strongest interactions were detected among the DAMs in Module 4, which included flavonoids and lipids, while among DEGs, the strongest interactions were observed in Module 2, which included genes belonging to GO classes such as: response to biotic stimulus, oxidoreductase activity and lipid metabolic process. Although Module 1 was only slightly less correlated within the module. However, the inter-modular interactions DEG-DEG and DAM-DAM were relatively weak. The correlations between the DAMs and DEGs were more complex and extended beyond the modules. The module with the most DAMs was Module 2. Many of these DAMs were correlated with one DEG from Module 3 encoding an early light-induced protein (*SECCE1Rv1G0044910*; ELIP). ELIP (being the intergroup hub of Module 3) was more strongly correlated with other compounds than gene encoding ADP-ribosylation factor (*SECCE1Rv1G0063470*). The DAMs associated with *ELIP* included apigenin glycoside and 5-hydroxyindoleacetaldehyde. In contrast, only a few DAMs in Module 3 (e.g., phenylpropanoid derivatives, including syringetin hexosides, rosmarinic acid hexoside, sinapaldehyde glucoside, and (epi)gallocatechin) were connected to several DEGs from Module 1. There were strong correlations between the DAMs from Module 1 (e.g., feruloylphenyllactic acid isomers) and the DEGs from Module 2, including genes encoding teosinte branched 1 (*SECCEUnv1G0548560*) and kaurene synthase (*SECCE7Rv1G0520210*). Gen *SECCEUnv1G0548560* was both the hub and intergroup hub of Module 2. In addition, this module contained six other intergroup hubs (including the *SECCE7Rv1G0520210* gene). Only one DEG, which encodes glutathione S-transferase (*SECCE4Rv1G0282280*), in Module 4 was linked with the Module 4 DAMs, of which lipidsoleic acid from lipids and *p*-coumaroylquinic acid and myricetin *O*-hexoside from phenylpropanoids and flavonoids, respectively, were identified. Neither Module 2 nor Module 4 among the DAMs had intergroup hubs.

Our analysis reveals a complex network of gene and metabolite interactions and revealed both strong intra-module interactions and the more complex inter-module relationships.

## Discussion

Despite many years of research, the genetic basis of rye resistance to LR is still poorly understood, with the available information mostly derived from Mendelian-based analyses [4, 5]. The recent sequencing of rye genomes [6, 7] and the publication of two articles describing the molecular basis of the rye immune response to the pathogen responsible for LR [8, 9] have verified the findings of earlier related research, while also further elucidating the rye response to LR. Specifically, several QTLs and regions containing NLR-encoding gene clusters were identified in the Lo7 genome, two of which include *Pr* genes (*Pr3* on 1RS and *Pr6*, which is similar to wheat *Lr1* in terms of the encoded protein). Unfortunately, the gene functions have not been confirmed at the transcriptome or metabolome level. That’s why, we decided to combined transcriptomic and metabolomic analyses supported by microscopy-based examinations of plant–pathogen interactions to more comprehensively characterize the mechanisms mediating rye defenses against LR.

This study involved three highly inbred rye lines (D33, D39, and L318) that were previously analyzed in terms of their immune response to LR under field and laboratory conditions. However, in earlier studies, these lines were either infected with a mixture of LR isolates present at a given time and at a given location [11] or with the isolate associated with the most uniform host–pathogen interaction among all tested rye lines [10]. Therefore, we used four carefully selected *Prs* isolates to investigate the rye responses to compatible and incompatible *Prs* isolates.

### Phenotyping of the rye-*Prs* interaction

The specificity of the plant–pathogen interaction was clarified on the basis of microscopic and macroscopic examinations, which enabled the classification of compatible and incompatible plant–pathogen interactions. In the compatible interaction, there was unrestricted pathogen growth, while in the incompatible interaction, micronecrosis were observed, indicative of the inhibitory effects of the plant on pathogen growth. The earlier micronecrosis in line D39 than in line D33 was suggestive of a more effective inhibition of pathogen growth, which was confirmed by the evaluation of the infection types (Fig. 2A). These observations correlate very well with results in the wheat – leaf rust interaction, where micronecrosis was observed very early in highly resistant lines (Tc*Lr9* or Tc*Lr26* - infection type 0), while micronecrosis did not occur in the compatible interaction (Thatcher – infection type 4) [52]. Necrosis, described as hypersensitive cell death, were also observed in the wheat – yellow rust interaction in both incompatible and compatible interactions [53]. This reaction was earlier and more extensive in incompatibles, whereas in compatible it was few and only at the initial stage of infection, as in compatible interaction D33 (72 hpt) in our experiment. Observed micronecrosis in an incompatible reaction is a hypersensitivity reaction that is crucial for effective resistance [54].

### Hallmarks of LR revealed by a transcriptome analysis

The changes in the rye transcriptome following an infection by *Prs* were detected using an RNA-seq approach. In recent years, RNA-seq analyses have become common in various biological research fields. For example, it has been used to identify plant resistance genes [14, 15]. To identify the significant DEGs, we analyzed the transcriptomic data using the following strict selection criteria to eliminate the genes with only a minor role in the rye defense response to LR: FDR < 0.01, |log_2_(fold-change)| ≥ 2, and BM > 50. Although transcriptome analyses are typically conducted using less restrictive criteria (e.g., FDR < 0.05 and |log_2_(fold-change)| ≥ 0.5–1) [e.g. [14, 15, 55]], some previous studies used similarly strict selection criteria. For example, Coram et al. [56] used strict selection criteria to analyze the transcripts associated with race-specific resistance to stripe rust in wheat. Furthermore, a third parameter (i.e., BM) must also be considered, but it is often overlooked despite its importance for characterizing the expression level of a specific gene. We believe that in certain instances, DEGs selected solely on the basis of other parameters may be unreliable and lack genuine biological significance. The proportions of the up-regulated and down-regulated DEGs were almost the same (52% vs. 48%). In contrast, only 15% of the identified DEGs were down-regulated in the wheat–*Agropyron cristatum* 2P addition line infected with LR in a recent study [15]. The proportions of up-regulated and down-regulated DEGs varied among the three rye inbred lines, with up-regulated genes representing the main group of DEGs in line D39, whereas the opposite trend was detected for line D33 (even for the infection with ICP *Prs*). For line L318, there were more up-regulated DEGs than down-regulated DEGs, but the difference was less than that detected in line D39. Poretti et al. [14] observed that LR infections down-regulate the expression of numerous genes in susceptible lines. In our previous studies, D33 and L318 were respectively the most and least resistant lines, while D39 exhibited an intermediate resistance, following an infection with a semi-compatible *Prs* isolate under field [11] and laboratory conditions [10]. However, in the current study, these lines responded differently to compatible and incompatible *Prs* isolates. This difference may be related to changes in sensitivity due to inbreeding; in the last few years, there has been a progressive weakening of line D33 under field conditions. Similar inbreeding depression has not been observed in the other two lines. Notably, only two NBS-encoding genes were identified using our strict selection criteria. Specifically, *SECCE6Rv1G0420800*, which was localized to chromosome 6R and encodes an NBS-LRR disease resistance protein, was detected in the L318_CP_36 comparison, whereas *SECCE7Rv1G0523960*, which was localized to chromosome 7R and encodes an NB-ARC domain-containing protein, was detected in the D39_CP_20 and D39_CP_36 comparisons. Both genes were differentially expressed at a relatively low level in the compatible plant–pathogen interaction. Perhaps *SECCE6Rv1G0420800* is co-localized with the *Pr1* or *Pr-e* genes, while *SECCE7Rv1G0523960* is co-localized with *Pr2* previously identified by Wehling et al. [2] and Roux et al. [4], but this possibility will need to be experimentally verified. None of the genes detected in our study matched the genes identified by Vendelbo et al. [8, 9]. However, the DEGs identified using less stringent parameters 1 ≥ |log_2_(fold-change)|≤ 2, which are not provided herein, included *SECCE5Rv1G0365570*, which was detected by Vendelbo et al. [9] at position 807.97 Mb on chromosome 5RL. This gene was differentially expressed exclusively in line D33 infected with ICP *Prs*, but only at 36 hpt. The expression of NBS-encoding genes at low levels does not reflect the contributions of these genes to the rye immune response to *Prs* [19], particularly during the development of ETI.

### Most common genes associated with the interactions between rye and CP and ICP isolates

Among the 877 unique DEGs, three groups of genes encoding CYP450s, RLKs, and POs, were more abundant than the other genes. The enzymes encoded by these genes are the primary contributors to plant immune responses to fungal pathogens, including *Puccinia* species [10, 57, 58]. Accordingly, their presence among the DEGs affected by *Prs* was unsurprising.

The CYP450s, which belong to one of the largest enzyme families, are crucial facilitators of NADPH- and/or O_2_-dependent hydroxylation reactions in primary and secondary metabolism across various organisms. In plants, they are also critical for responses to abiotic and biotic stresses because they affect phytoalexin biosynthesis, hormone metabolism, and the biosynthesis of some other secondary metabolites such as BXs [59, 60]. Dobon et al. [61] reported that the expression of CYP450-encoding genes increases in wheat infected with *Puccinia striiformis* f. sp. *tritici* at 3 dpi, which coincides with the timing of haustorium proliferation. In our study, two CYP450-encoding genes (*SECCE4Rv1G0248210* and *SECCE7Rv1G0520230*) were common among the DEGs in all three inbred lines. The first gene (*SECCE4Rv1G0248210*; up-regulated mostly after the CP *Prs* infection) encodes dolabradiene monooxygenase, which converts dolabradiene to dolabralexins, a class of defense-related diterpenoids. These compounds accumulate in maize treated with the fungal pathogens *Fusarium verticillioides* and *Fusarium graminearum* [62]. In addition to maize, dolabradiene monooxygenase has been detected in only a few coniferous tree species in the families *Araucariaceae* and *Cupressaceae*. The identification of *SECCE4Rv1G0248210* as a DEG in all examined rye lines indicates that dolabralexins are synthesized by rye and these specialized diterpenoid metabolites may participate in the immune response of rye to *Prs*. There is no information available regarding the specific function of the second CYP450-encoding gene (*SECCE7Rv1G0520230*) during the response to *Prs*. We previously determined that the expression levels of two other CYP450-encoding genes, namely *SECCE5Rv1G0298500* (*ScBx4*) and *SECCE5Rv1G0298490* (*ScBx5*), are down-regulated by LR [10]. The RNA-seq analysis conducted in the current study indicated the expression of these genes was down-regulated by LR, but only in the following four comparisons: D33_CP_36 and D33_ICP_36 (*SECCE5Rv1G0298500*) and L318_CP_20 and L318_CP_36 (*SECCE5Rv1G0298490*). However, in line L318 infected with the ICP isolate, *ScBx5* expression was up-regulated. This may be due to the highest DIBOA content in the L318 line (compared to the other two lines; Table S1), in whose biosynthesis the *ScBx4* gene is involved. Besides *ScBx4* and *ScBx5* genes, another gene controlling BX biosynthesis detected by us previously [10], namely *Scglu* (*SECCE2Rv1G0138870*), was also identified in the RNA-seq analysis as a downregulated DEG, only in the D33 line.

The receptor-like kinases (RLKs) serve as pattern recognition receptors that perceive signals or specialized elicitors secreted by pathogens known as PAMPs, thereby activating PTI [63]. Therefore, it is somewhat surprising that RLK-encoding genes have not been the subject of transcriptomic studies on LR or other rusts. There are only a few published articles on this topic. According to RNA-seq analysis performed by Zou et al. [58], in *Triticum urartu* infected with stripe rust, the *TuRLK1* expression level increases. The encoded RLK is essential for the immune response to stripe rust, which is mediated by the NLR protein YrU1. Additionally, Gu et al. [64] investigated the role of the cysteine-rich receptor-like kinase (CRK), which belongs to a large subgroup of plant RLKs. They focused on *TaCRK2* and its expression during an incompatible interaction with LR, which is dependent on Ca^2+^. By decreasing *TaCRK2* expression in wheat, they observed a dramatic increase in the hypersensitive response and the number of HMCs at the infection site. Considering the role of RLKs in immune responses, one might expect that the expression of the corresponding genes would increase in LR-infected rye. The up-regulated expression of RLK-encoding genes was generally observed in two lines (D39 and L318) infected with the compatible isolate of *Prs*. However, in D33, only three of the 10 RLK-encoding genes had up-regulated expression levels, which may help to explain the weak defensive potential of this line. This may be related to its extensive inbreeding.

The significance of POs in the induced plant defense against fungal pathogens is associated with their role in reinforcing the cell wall (i.e., physical barrier) and enhancing the production of ROS and phytoalexins [65]. The relationship between stem rust resistance and increased PO activity was first reported in 1971 [66]. However, there have been relatively few published reports describing this relationship. Nevertheless, earlier research confirmed that the total [67] and intercellular PO [68] activities increase in response to LR. Dmochowska-Boguta et al. [57] observed that two of the four POs in wheat are strongly induced by LR. Moreover, in the susceptible cultivar Thatcher and resistant isogenic lines with different *Lr* resistance genes, there is a PO-dependent oxidative burst. It was suggested that (class III) POs play a leading role in ROS formation during the wheat response to LR. The importance of POs in immune responses was also demonstrated in an earlier study on stem rust-infected wheat by Moerschbacher et al. [69]. More specifically, PO activities increased in infected wheat plants (compatible and incompatible interactions) from 16 to 48 hpt; after this period, the PO activity in the resistant plants continued to increase for up to 7 days (compatible interaction). Alternatively, the PO activity either remained constant or slowly decreased beginning at 2 dpi (incompatible interaction) [69]. In our analysis, the expression of most PO-encoding genes was up-regulated (mostly after the CP *Prs* infection at 36 hpt); two of these genes were also expressed at very high levels at 36 hpt in line L318. Almost all cases of down-regulated *PO* expression were detected in line D33.

In addition to the groups discussed above, we detected several others that were more specific either for a given type of plant–pathogen interaction and/or for a given time-point. Some of the genes that were primarily induced by CP *Prs* were revealed to encode methylesterases (MEs), which are essential enzymes that coordinate carbohydrate metabolism, stress responses, and sugar signaling [70]. Following an infection by fungal pathogens, methyl esterifications can improve plant resistance because highly methyl-esterified pectins may be less susceptible to the hydrolytic activities of pectic enzymes, including fungal endopolygalacturonases [71, 72]. Wiethölter et al. [73] demonstrated that during a stem rust infection of wheat, there is a significant difference in the homogalacturonan contents between susceptible and resistant lines. This difference is associated with a nonrandom and blockwise distribution of the MEs in the susceptible lines, which is in contrast to the more random distribution of these enzymes in the resistant lines. In the present study, only two ME-encoding genes (*SECCE3Rv1G0211990* and *SECCE3Rv1G0211270*) were common DEGs in all three rye inbred lines. In D33 and L318, the expression of these genes was up-regulated. Unexpectedly, the expression levels of these genes were down-regulated at 36 hpt in D39 infected with compatible isolates. Assuming that methyl esterifications enhance plant resistance, it is reasonable to expect the expression of ME-encoding genes to increase, at least during incompatible interactions. We observed that in line D39, in which the majority of genes relevant to defense responses to LR were up-regulated, these two genes were down-regulated. Interestingly, only one gene (*SECCE3Rv1G0193070*) from the ME family was up-regulated at 20 hpt in D33 infected with the ICP isolate. Nevertheless, in *Blumeria graminis* f. sp. *hordei*, thiopurine methyltransferase may be targeted by a fungal effector candidate [74, 75]. Hence, it remains possible that MEs play a significant role in the rye–*Prs* interaction. Our transcriptome analysis revealed a significant decrease in *SECCE7Rv1G0457810* expression in all inbred lines infected with a CP isolate, implying the encoded enzyme may be targeted by *Prs*.

By analyzing our RNA-seq data, we identified pathogenesis-related protein 1 (PR-1)-encoding genes as the only large group of genes with up-regulated expression levels at 20 hpt in the samples infected with both CP and ICP isolates. The PR-1 proteins can inhibit the growth of a variety of fungal pathogens [76]. In wheat, two *PR-1* genes, namely *TcLr19PR1* [77] and *TaLr35PR1* [78], confer resistance to LR. Neugebauer et al. [79] determined that increased *PR-1* expression (Acc. No FJ815167) is related to the immune response of the susceptible cultivar Teacher to LR infections. In our analysis, three PR-1-encoding genes (*SECCE5Rv1G0309790*, *SECCE5Rv1G0309810* and *SECCE5Rv1G0309830*) were expressed at high levels in both types of plant responses to *Prs*. The expression of these three genes was up-regulated at 20 hpt in D39 infected with CP and ICP isolates, indicating they may be involved in the initial activation of plant defense mechanisms. Additionally, pathogenesis-related protein 1 (*SECCE7Rv1G0464120*) expression was up-regulated regardless of the response type in both D33 and D39. Notably, the magnitude of the expression changes was greater in D39 than in D33. In contrast, in L318, the expression of only two PR-1-encoding genes was up-regulated at 36 hpt, namely *SECCE5Rv1G0359230* [log_2_(fold-change) of 2.05] and *SECCE7Rv1G0480890* [log_2_(fold-change) of 2.79]. Interestingly, in D39 infected with CP *Prs*, the expression levels of the genes encoding PR-1 proteins were mainly up-regulated, but almost exclusively at 20 hpt.

The genes encoding chlorophyll *a*/*b*-binding proteins (CabBP) had a specific down-regulated expression pattern in line D33, especially at 20 hpt in the plants infected with CP isolates. This down-regulated expression was observed for 10 genes. In contrast, at the same time-point during the ICP infection, the expression of only one gene (*SECCE3Rv1G0200810*) was down-regulated in D33. In line L318 infected with the CP isolate, the expression of only one gene from the *CabBP* family was up-regulated (*SECCE4Rv1G0250540*) at 36 hpt. Unexpectedly, there were no significant DEGs related to CabBP in D39. Earlier research confirmed CabBPs, which are representative nuclear-encoded chloroplast proteins, are components of the light harvesting complex of photosystem II and are present in the thylakoid membrane of photosynthesizing plants [80]. By providing energy, photosynthesis is closely integrated into the defense response to pathogens [81]. The suppression of nuclear-encoded chloroplast proteins may allow pathogens to overcome PTI [82]. However, there is currently no evidence of a relationship between these proteins and rust resistance. Thus, to the best of our knowledge, this is the first study to show that LR significantly inhibits the expression of CabBP-encoding genes in a genotype-dependent manner.

The xyloglucan endotransglucosylase/hydrolases (XTHs) belong to a group of enzymes specific to plant–pathogen interactions [83–85]. The expression of XTH-encoding genes was down-regulated following the infection with ICP *Prs*. These genes were primarily expressed in line D33 at 36 hpt. The functions of these enzymes related to the immune response to certain fungal pathogens have been thoroughly characterized. By catalyzing the cleavage and polymerization of xyloglucan molecules, XTHs mediate cell remodeling and are considered to be key enzymes for plant cell wall reconstruction [83–85]. Thus, the roles for XTHs during responses to cell wall-degrading pathogens seem obvious. Indeed, the protective effects of XTHs have been confirmed in plants infected with certain fungal pathogens, including *F. graminearum* [86], *Pyrenophora teres* [87], and *Macrophomina phaseolina* [88]. The observed down-regulated expression of XTH-encoding genes was in accordance with the findings of earlier studies on the expression of these genes. There is currently no evidence of an association between the increased accumulation of XTHs and the increased expression of the related genes and immune response to LR.

The glycosyltransferases (UGTs) are enzymes belonging to a multigenic and highly diverse superfamily that is ubiquitous among living organisms and is associated with disease resistance, including LR resistance. According to Pujol et al. [89], *Ta.90050*, which encodes a UGT, is putatively involved in the late resistance response of wheat to stem rust. In our RNA-seq analysis, we identified two UGT-encoding genes, which had down-regulated expression levels specifically in D33. The expression of the first gene (*SECCE7Rv1G0520220*) was down-regulated during the interaction with the CP isolate, while the expression of the second gene (*SECCE6Rv1G0435050*) was down-regulated during the interaction with the ICP isolate. Recently, Amo and Soriano [90] used a meta-QTL analysis approach to identify five up-regulated genes encoding GTs, of which *TraesCS7D02G217700* was proposed as a candidate gene mediating LR resistance. In rye, the protective role of GTs may be associated with the fact they catalyze the conversion of DIBOA to DIBOA glucoside, which accumulated in two lines (D39 and L318) between 8 and 24 hpt and in line D33 at 24 hpt in plants inoculated with the semi-compatible *Prs* strain [10]. Our RNA-seq data analysis indicated the up-regulated expression of GT-encoding genes generally occurred at 36 hpt. However, these genes were not specific to any of the analyzed inbred lines or reaction types (compatible or incompatible). Because UGTs contribute to the biosynthesis of cell wall polysaccharides and glycoproteins, these genes may be important for plant defense responses to pathogens, especially considering LR-induced plant cell wall modifications are essential for HMC development.

Antifungal hydrolases (beta-1,3-glucanase; Glu) belonging to the PR-2 family reportedly influence plant defense responses to fungal pathogens, including those responsible for LR [77, 79, 91, 92], stem rust [93], and stripe rust [94]. Münch-Garthoff et al. [93] observed that the activation of *glu* transcripts occurs very early, approximately 16 h before the typical hypersensitive response is detectable, which is long before a tight contact between the pathogen and a host cell is established. In wheat, the expression of the Glu-encoding gene *TcLr19Glu* is induced by *Pt* during both compatible and incompatible interactions, but the expression levels are greater during incompatible interactions. The *TcLr19Glu* expression level peaks between 24 and 48 hpt [77]. Neugebauer et al. [79] analyzed wheat cultivar Thatcher infected with six *Pt* races. They detected a gradual increase in the expression of a Glu-encoding gene as well as PR-1 and PR-5 thaumatin-like protein-encoding genes between 1 and 3 dpi, which was followed by a decrease in expression until 5 dpi and then another increase at 6 dpi. This may indicate that specific changes in the production of beta-1,3-glucanases influence whether an LR infection is successful. During our transcriptomic analysis, we detected fluctuations in *Glu* expression. For example, in D39 infected with the CP isolate, the log_2_(fold-change) for *SECCE6Rv1G0429310* was 3.18 at 20 hpt, whereas it was 2.15 at 36 hpt. In contrast, the log_2_(fold-change) for *SECCE6Rv1G0429650* in D39 during the compatible interaction was 2.62 at 20 hpt and 2.83 at 36 hpt. This trend was even more pronounced for *SECCE6Rv1G0429680*, with a log_2_(fold-change) of 2.09 and 2.89 at 20 and 36 hpt, respectively. Several genes encoding beta-1,3-glucanases had up-regulated expression levels, particularly during the compatible interactions involving line D39.

Although trypsin inhibitors (TIs) are protease inhibitors that are among the first PTI-related proteins to be activated in response to pathogens [95, 96], their relationship to rust resistance is unknown. The only article describing the involvement of serine-type protease inhibitors [96] indicated that in wheat plants infected with stripe rust, several genes encoding Bowman-Birk (BBI) protease inhibitors are differentially expressed (usually up-regulated). The TI encoded by *SECCE5Rv1G0365990*, which was selected on the basis of our RNA-seq data, belongs to the BBI class. The genotype specificity of the expression of this gene may be indicative of a role in the general plant response to LR.

The above-mentioned findings highlight the complexity of the response of rye inbred lines to LR, while also emphasizing the importance of interpreting the results on the basis of the *Prs* isolate, time-point, and genetic background of the plant.

### Common and specific transcripts among the CP and ICP interactions

Fifteen DEGs were common to all comparisons (Fig. 4B; Table S5). These DEGs may encode proteins with critical effects on pathogen development regardless of the reaction type, implying they are “core genes” for the rye response to LR. Interestingly, the expression levels of all of these genes were up-regulated by *Prs*. Some of these genes encode proteins belonging to the CYP450, endo-1,3-beta-glucanase, DXS, ammonium transporter, and aldo/keto reductase families. Moreover, three of these genes belong to the WIR1a family. The members of this family encode integral membrane proteins affecting the cell wall structure [97] and immune responses to fungal pathogens, including those causing rust infections. As mentioned above, Coram et al. [56] determined that among the 28 stripe rust-induced genes at 24 and 48 hpt, the gene encoding a pathogen-induced WIR1A protein is the fourth most important gene for plant responses to stripe rust (i.e., after the genes encoding a copper-binding protein, heat-stress transcription factor, and kaurene synthase). In a previous study by Chen et al. [98], the common wheat genes associated with the *Yr39*-mediated adult-plant resistance to high temperatures and the *Yr5*-mediated all-stage resistance included a WIR1-encoding gene. In plants, DXS is an essential enzyme for isoprenoid biosynthesis because it catalyzes the conversion of pyruvate and glyceraldehyde 3-phosphate to 1-deoxy-D-xylulose 5-phosphate, which is a key precursor of important plant isoprenoids, including carotenoids, chlorophylls, gibberellins, and essential oil constituents [99]. However, there is no evidence indicating any association between this specific cluster of genes and the response to LR.

Ammonium transporters primarily facilitate the uptake, distribution, and homeostasis of ammonium (NH_4_^+^) in plants, but their specific involvement in LR infections is unclear. Jiang et al. [100] detected the up-regulated expression of an AMT2-type ammonium transporter gene (*TaAMT2;3a*) in wheat infected with a virulent *Prs* isolate. Moreover, *Prs* growth is hindered by a decrease in *TaAMT2;3a* expression, resulting in a decrease in the number of hyphal branches and HMCs.

In the immune response to LR, differentially regulated genes, specific only for a given line and/or a given type of interaction, are equally important. Our analyzes detected four groups of such genes encoding NAC domain-containing protein, specific for CP, XTH specific for ICP, CabBP and Aquaporin, differentially regulated only in line D33. The role of two of the above-mentioned proteins - XTH and CabBP in the immune response to LR has been discussed above. The functions of the other two proteins are also related to this reaction.

The plant NAC gene family has been suggested to play important roles in stress response [101]. For LR, the role of these proteins has been proven in wheat [102]. Using the same experimental approaches as we have - RNA-seq and RT-qPCR, the authors identified the activation of the *TaNAC069* gene in response to *Puccinia triticina* and related signaling molecules. Aquaporins are membrane channel proteins present in all living organisms and having many physiological functions during plant growth and development. They are assumed to play also an important role in plant defense responses against biotic and abiotic stresses including fungal diseases [103]. Among wheat genes affected by LR, Prasad et al. [104] found genes encoded aquaporins. However, its expression was the highest at the pre-haustorial stage (6 and 12 h post inoculation), so much earlier than in our experiment.

### KEGG enrichment analysis *in silico*

The KEGG pathway enrichment analysis enabled us to identify specific categories that were associated with different time-points and reaction types, thereby providing insights into the plant defense response. At 20 hpt of the CP interaction, the enrichment of “Carbohydrate metabolism” and “Energy metabolism” suggests that these processes play a role in plant defense mechanisms. It can be assumed that the synthesis and use of carbohydrates and energy are crucial for sustaining the metabolic requirements associated with an effective defense against pathogens [105]. Interestingly, in the ICP reaction at 20 hpt, a more general category (“Metabolism”) was enriched, implying that various metabolic pathways may be activated during the plant defense response, which reflects the complexity and the interconnected nature of plant immune mechanisms. Furthermore, the enrichment of “Genetic information processing” during the CP and ICP interactions suggests that transcriptional reprogramming is critical for plant immune responses to LR.

At 36 hpt during the CP interaction, the enrichment of “Metabolism of cofactors and vitamins” and “Organismal systems” is indicative of the activation of additional defense mechanisms. These categories encompass several processes, such as the production of secondary metabolites, reinforcement of physical barriers, and modulation of overall physiological responses, that enhance plant resistance to pathogens. Phenylpropanoid metabolism is considered to be one of the most important metabolic processes in plants. For example, it influences the interaction between plants and the environment by providing flavonoids that “scavenge” the ROS induced by environmental stresses and many defense-related specialized metabolites [15, 20, 21, 106, 107]. Phenylpropanoid metabolism is also important for the defense response to LR. The KEGG analysis and GSEA performed by Ji et al. [15] assigned the DEGs in wheat–*Agropyron cristatum* 2P addition line II-9-3 infected with LR to several pathways (e.g., phenylpropanoid biosynthesis). In our metabolomic analysis, phenylpropanoids were identified as DAMs in most comparisons (discussed later). Furthermore, Tsers et al. [17] showed that the initial reactions of the phenylpropanoid biosynthesis pathway may be induced in rye infected with *M. nivale*. Conversely, during the ICP reaction, the enrichment of “Amino acid metabolism” and “Energy metabolism” at 36 hpt suggests that the plant intensifies its metabolic activities to cope with the ongoing defense response. The synthesis and utilization of amino acids and energy-rich molecules are likely vital for satisfying the heightened metabolic demands associated with a successful defense against pathogens [108].

In summary, our KEGG pathway enrichement analysis identified genes involved in carbohydrate and energy metabolism that are specific to the CP interaction. The enrichment of diverse metabolic categories in the ICP interaction may reflect the activation of a multifaceted defense response, potentially involving the production and use of secondary metabolites. Additionally, the enrichment of “Genetic information processing” and various metabolic categories is indicative of the activation of complex defense mechanisms and transcriptional reprogramming during both compatible and incompatible interactions with *Prs*.

### High genotype- and treatment specificity of the metabolome-related immune response

In our metabolomics studies we found a predominance of genotype- and treatment specific treatment-specific DAMs over common DAMs in all comparisons. The genotype-specific DAMs may be indicators of the intra-species diversification of immune responses, making them potentially useful metabolic biomarkers of LR resistance that can optimize the selection of the most resistant cultivars [107]. Among the tested lines, the line L318 had the most distinct immune response to both *Prs* strains, which may be related to its relatively low resistance [109].

We identified several DAMs characteristic of both types of interactions: for the infection with the CP isolate benzoic acid derivatives and flavonoids were most specific when for ICP – these were chrysin and peptides (such as isoleucylglutamine, isoleucylvaline and S-adenosyl-homocysteine). The role of benzoic acid derivatives and flavonoids in the immune response against fungal pathogens, including rust fungi, is well known [109]. However, Mashabela et al. [109] showed that in wheat infected with *Puccinia striiformis* f. sp. *tritici* both defence metabolites are accumulated faster in resistant cultivar (ICP interaction) compared to the susceptible (CP interaction) cultivar. So it seems that rye has its own rye-specific way of defending against LR. To our knowledge, the role of chrysin, isoleucylglutamine, isoleucylvaline and S-adenosyl-homocysteine in plant defense against LR has never been investigated. So, we are the first to describe these compounds as defense metabolites synthetized by rye in response against LR, and, additionally, specific for ICP interactions.

### Complex relationships between the rye transcriptomic and metabolomic responses to LR

The significant correlations among the DAMs and DEGs reflected the extensive reprogramming of rye metabolic activities during an infection with *Prs*. The functional annotation of the DAMs and DEGs revealed pathways mainly related to the modulation of ROS levels. The dominant effects of phenylpropanoids in the metabolomic response of cereals infected with *Puccinia* ssp. were previously noted for wheat [32], maize [110], and barley [111]. Metabolites related to the “Phenylpropanoid biosynthesis” pathway are the precursors for specialized compounds that scavenge ROS, regulate photosynthesis, and inhibit fungal growth, thereby influencing plant immunity [20, 112]. Multiple roles for phenylpropanoids in plant defense responses are consistent with the reported association between cereal stress resistance and hydroxycinnamic acid derivatives esterified with amides [21, 106] and quinic acids [30]. In plants, phenylpropanoids are produced as soluble compounds or as cell wall components; the analysis of the latter compounds requires an alkaline extraction step. Therefore, only soluble metabolites were considered in the current study [21]. The identification of phenylpropanoids with multiple functions is consistent with our detection of a strong interaction between pathogen-triggered metabolites and genes. Such complex relationships presumably reflect the diversity in the metabolic mechanisms associated with these compounds. Intriguingly, the phenylpropanoids assigned to “Flavone and flavonol biosynthesis” and “Flavonoids biosynthesis” accumulated exclusively at 36 hpt in lines D33 and L318 infected with the ICP isolate.

The induction of “Ascorbate and aldarate metabolism” as an immune response in L318 infected with the ICP isolate was indicative of the accumulation of effective antioxidants as well as regulators of photosynthesis and transmembrane electron transport [113]. Another effective antioxidation system related to “Glutathione metabolism” was detected in D33.

There was also a considerable enrichment of the diterpenoid-related metabolic pathways in rye infected with *Prs*. Diterpenoids contribute directly to plant defenses against pathogenic fungi through their antibiotic activities [114]. The accumulation of many terpenoid compounds in rice, such as oryzalexins and momilactones, is positively correlated with increases in the efficiency of basal defense responses in rice [27]. The current study revealed the relationships between plant immunity and gibberellins, which are mainly known as regulators of developmental processes throughout the plant life cycle. Nevertheless, gibberellins contribute to plant immunity by modulating the SA/JA cross-talk in the immune signaling network as well as the scavenging of ROS [115]. In rice, the accumulation of gibberellins increases the resistance to necrotrophs and the susceptibility to (hemi)biotrophs [116]. Interestingly, the opposite effects were observed in wheat and barley [117]. In our experiments, the metabolites in the gibberellin biosynthesis module mainly accumulated in response to both types of *Prs* strains, suggestive of the complex effects of gibberellins on rye immune responses.

The observed increase in the contents of the carbohydrates associated with “Starch and sucrose metabolism” in D33 infected with the CP isolate is likely related to a disturbance in sugar homeostasis and their storage [118]. Starch and sucrose metabolism is closely related to soluble, galactose-derived oligosaccharides, which are predicted to be antioxidants and ABA-related signaling molecules [119]. Moreover, the management of sugar levels modulates the expression of defense-related phenylpropanoids [118]. Therefore, we speculate that the enrichment of these pathways is associated with the regulation of secondary metabolites. This is supported by the findings of an earlier study, which indicated biotrophic pathogens consume significant amounts of carbohydrates from the host plant, which disrupts normal carbohydrate and nucleotide sugar metabolism [120]. The enrichment of “Cutin, suberine and wax biosynthesis” during the interaction between D33 and the ICP isolate may be related to the reinforcement of the extracellular barrier by cutin and suberin during pathogen infections [121]. In addition, similar to our results, a previous study involving wheat detected changes to the nitrogen reservoir induced by developing pathogens [122].

## Conclusions

In summary, our transcriptomic and metabolomic analyses identified multiple DEGs and DAMs following a *Prs* infection. This study is the first to apply such a comprehensive approach to examine LR and rye. Additionally, the importance of some genes for the immune response of rye to LR, such as genes encoding dolabradiene monooxygenase, thiopurine methyltransferase, XTH, and basic secretory proteins, was revealed for the first time. This research is an important step toward understanding the early reaction of rye to an infection with *Prs*, which is responsible for one of the most damaging rye diseases including identification of both common and specific DEGs and DAMs for CP and ICP interaction. Likewise, a pool of genotype-specific and common DEGs for all three rye lines was identified. The discrepancy found in the dynamic changes of DEGs and DAMs between ICP and CP interactions suggests that there is a complex response that leads to plant resistance or susceptibility. Using various research approaches we were able to unveils an intricate network of gene and metabolite interactions, providing insights into potential key regulatory components. This insight is derived from the identification of strong intra-module interactions as well as more complicated inter-module relationships. Furthermore, the generated data may form the basis of genome- and metabolome-based selection to support rye breeding toward increasing phenylpropanoids, flavonoids, terpenoids and gibberellins accumulation as well as expression of genes encoding several more important groups of proteins, such as UGTs, beta-1,3-glucanases, cytochrome P450, PR-1 and POs.

### Supplementary Information

**Additional file 1: Table S1.** Characteristics of rye inbred lines, D33, D39, and L318, chosen for experiments.

**Additional file 2: Table S2.** Resistance reaction of three rye inbred lines, D33, D39, and L318, determined based on detached-leaf test.

**Additional file 3: Table S3.** List of primers used in RT-qPCR experiments.

**Additional file 4: Table S4.** log2(fold-change)|values for metabolites in all 12 comparisons (LC-MS). LC-MS data processed.

**Additional file 5: Table S5.** RNA-seq results for all DEGs (only comparisons containing DEGs are listed below). List of all unique DEGs. KEGG-based*) functional classification of all unique Differentially Expressed Genes (DEGs).

**Additional file 6: Table S6.** Common and unique DEGs for both types of interactions and both time points. Gene onthology analysis for group of common DEGs for both types of interactions and both time points. Gene onthology analysis for group of unique DEGs for CP 20hpt. Gene onthology analysis for group of unique DEGs for ICP 20hpt. Gene onthology analysis for group of unique DEGs for CP 36hpt. Gene onthology analysis for group of unique DEGs for ICP 36hpt.

**Additional file 7: Table S7.** Common and unique DEGs; different comparisons.

**Additional file 8: Table S8.** The most strongly up- and down-regulated genes.

**Additional file 9: Table S9.** DEGs common for three rye inbred lines.

**Additional file 10: Table S10.** The complete joint-pathway enrichment of the DAMs and DEGs.

**Additional file 11: Table S11.** Nodes of the correlation network and their properties. Edges of the correlation network and their properties.

**Additional file 12: Table S12.** Common and unique DAMs selected on the basis of Venn Diagram.

**Additional file 13: Fig. S1.** Comparison between relative expression levels determined by RNA-seq and RT-qPCR analyses. The sequences of primers used in RT-qPCR analysis (including primers for reference gene) are listed in Table S3. The asterisk (*) indicate statistically significant difference with p < 0.05.

## Data Availability

The raw RNA-seq (fastq) data are deposited in the NCBI database (BioProject: PRJNA888031).
