# Supplementary material for: Leaf rust (Puccinia recondita f. sp. secalis) triggers substantial changes in rye (Secale cereale L.) at the transcriptome and metabolome levels
Source: BMC Plant Biol. 2024 Feb 13;24:107. doi: 10.1186/s12870-024-04726-0 (PMC10863301; doi:10.1186/s12870-024-04726-0)
Supplement: Supplementary file 2 — Additional file 2: Table S2. Resistance reaction of three rye inbred lines, D33, D39, and L318, determined based on detached-leaf test. [file 12870_2024_4726_MOESM2_ESM.docx]

**Table S2.** Resistance reaction of three rye inbred lines, D33, D39, and L318, determined based on detached-leaf test.

| *Prs* isolate | **1.1.6** | **81/r/5_5x** | **83/2/2.2_5x** | **88/o/1_5x** | 49.2.5 | 60/17/2.1_4x | 67/17/2.2.1 | 72/17_2.4 | 72/17_2.5 | 80/13_4x | 83/n/2.1_5x | 86/n/2.1_5x | 88/2/9.3_5x | 91/2/2.1_4x | 92/2/2.2_5x |
| --- | --- | --- | --- | --- | --- | --- | --- | --- | --- | --- | --- | --- | --- | --- | --- |
| Rye line | Reaction type | | | | | | | | | | | | | | |
| D33 | **2** | 3 | **4** | 4 | 2 | 4 | 1 | 3 | 3 | 3 | 2 | 4 | 3 | 4 | 4 |
| D39 | **1** | 1 | **4** | 2 | 4 | 1 | 3 | 2 | 3 | 2 | 2 | 3 | 1 | 3 | 3 |
| L318 | 3 | **3** | 4 | **4** | 4 | 4 | 4 | 4 | 4 | 4 | 4 | 4 | 4 | 4 | 4 |

*- infection types according to disease rating scale developed by Murphy (1935), isolates selected for the main experiment are marked in bold
