# Supplementary material for: Leaf rust (Puccinia recondita f. sp. secalis) triggers substantial changes in rye (Secale cereale L.) at the transcriptome and metabolome levels
Source: BMC Plant Biol. 2024 Feb 13;24:107. doi: 10.1186/s12870-024-04726-0 (PMC10863301; doi:10.1186/s12870-024-04726-0)
Supplement: Supplementary file 3 — Additional file 3: Table S3. List of primers used in RT-qPCR experiments. [file 12870_2024_4726_MOESM3_ESM.docx]

**Table S3.** List of primers used in RT-qPCR experiments.

| **Gene** | **Name** | **ID** | **Primer name** | **Sequence** | **Amplicon size** | **Ta** | **Tm** |
| --- | --- | --- | --- | --- | --- | --- | --- |
| GLU | BETA-1,3-GLUCANASE | SECCE6Rv1G0429310 | qPCR_Sc_GLU-Fwd | TACCAGAACCTGTTCGACGC | 246 bp | 60 ^o^C | 93,25 ^o^C |
|  |  |  | qPCR_Sc_GLU-Rev | GCCCTTCCTGTTCTCGTTGA |  |  |  |
| UGT | (UGP)-GLYCOSYLTRANSFERASE | SECCE7Rv1G0520220 | qPCR_Sc_UGT-Fwd | ATACGGATCCCAAGGACCGA | 76 bp | 60 ^o^C | 86,35 ^o^C |
|  |  |  | qPCR_Sc_UGT-Rev | CTTGCATTGAATGGACCTTACCA |  |  |  |
| PR-1 | PATHOGENESIS RELATED PROTEIN 1 | SECCE7Rv1G0464120 | qPCR_Sc_PR-1-Fwd | CTGTTTCGTCGCCAAGGAGT | 269 bp | 60 ^o^C | 88,63 ^o^C |
|  |  |  | qPCR_Sc_PR-1-Rev | CCTCCAGCACCTCCATCTTG |  |  |  |
| DXS | 1-DEOXY-D-XYLULOSE 5-PHOSPHATE SYNTHASE | SECCE1Rv1G0055210 | qPCR_Sc_DXS-Fwd | CTTACGAGGCTCCAGTCCAG | 120 bp | 60 ^o^C | 88,39 ^o^C |
|  |  |  | qPCR_Sc_DXS-Rev | GCGGGCATACTCATCCACTT |  |  |  |
| CYP450 | CYTOCHROMES P450 | SECCE4Rv1G0248210 | qPCR_Sc_CYP450no3-Fwd2 | GGCAAGGAAGACACCCTCAG | 72 bp | 60 ^o^C | 85,94 ^o^C |
|  |  |  | qPCR_Sc_CYP450no3-Rev2 | TGAAAATATATCAAAGATGACGGCG |  |  |  |
| ME | METHYLESTERASE | SECCE3Rv1G0211990 | qPCR_Sc_ME-1-Fwd | CGAGAGGCTGATTCTGGTCG | 159 bp | 60 ^o^C | 90,17 ^o^C |
|  |  |  | qPCR_Sc_ME-1-Rev | CTTCGCATGAACTCCTCGGT |  |  |  |
| **ADP**^*)^ | ADENOSINE DIPHOSPHATE | SECCE3Rv1G0194660 | qPCR_Sc_ADP-Fwd | AGCGTGTTGTTGAGGCTAGA | 183 bp | 60 ^o^C | 84,61 ^o^C |
|  |  |  | qPCR_Sc_ADP-Rev | AAGTGCTCTGAATGTACCAGTG |  |  |  |

^*)^ reference gene

Ta = annealing temperature

Tm = melting temperature
