# Supplementary material for: Leaf rust (Puccinia recondita f. sp. secalis) triggers substantial changes in rye (Secale cereale L.) at the transcriptome and metabolome levels
Source: BMC Plant Biol. 2024 Feb 13;24:107. doi: 10.1186/s12870-024-04726-0 (PMC10863301; doi:10.1186/s12870-024-04726-0)
Supplement: Supplementary file 13 — Additional file 13: Fig. S1. Comparison between relative expression levels determined by RNA-seq and RT-qPCR analyses. The sequences of primers used in RT-qPCR analysis (including primers for reference gene) are listed in Table S3. The asterisk (*) indicate statistically significant difference with p < 0.05. [file 12870_2024_4726_MOESM13_ESM.pptx]

## Slide 1
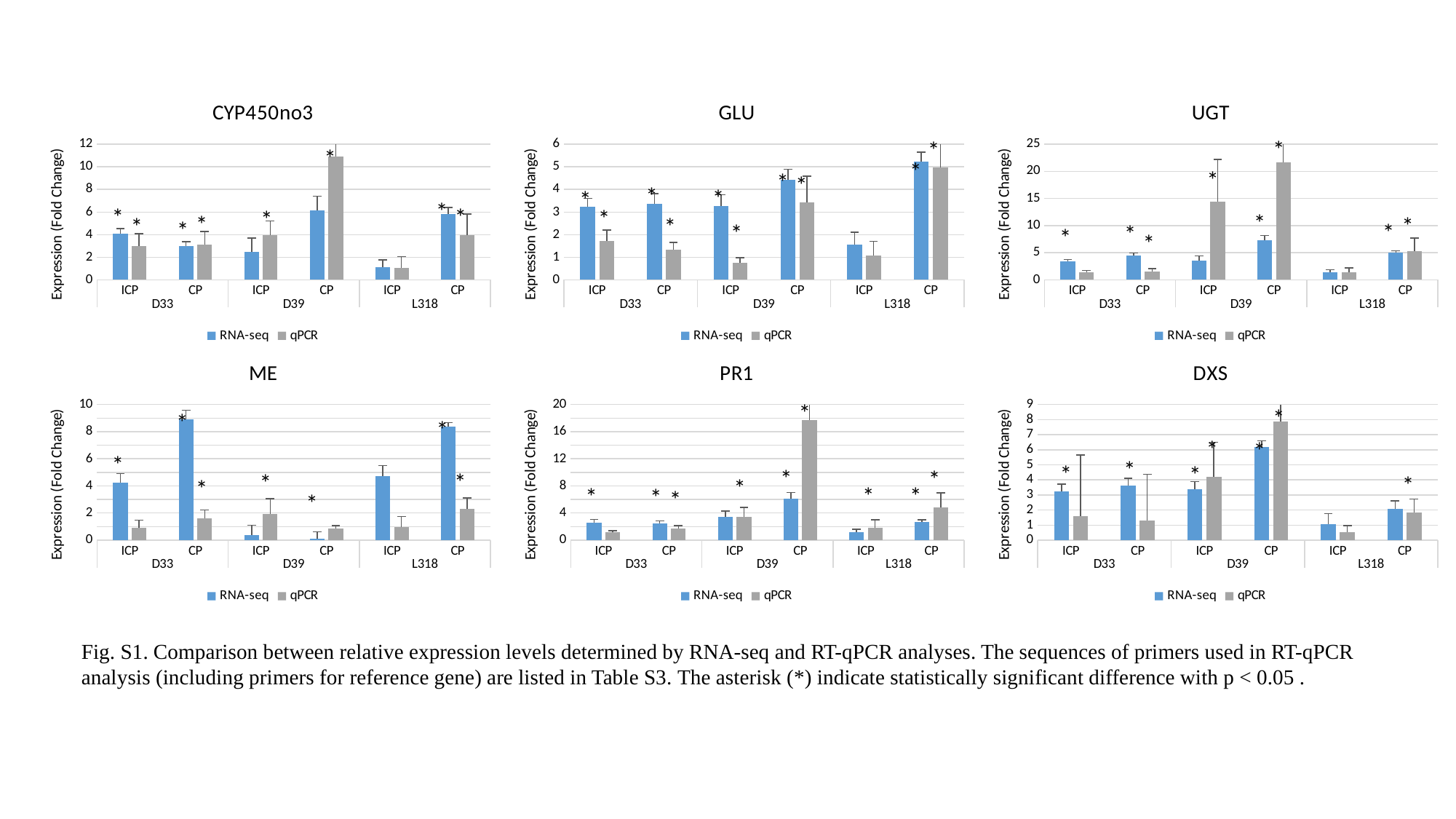

### Chart: CYP450no3
| Category | RNA-seq | qPCR |
|---|---|---|
| ICP | 4.11 | 2.966 |
| CP | 3.0 | 3.146 |
| ICP | 2.45 | 3.949 |
| CP | 6.16 | 10.928 |
| ICP | 1.11 | 1.062 |
| CP | 5.84 | 3.954 |
### Chart: GLU
| Category | RNA-seq | qPCR |
|---|---|---|
| ICP | 3.22 | 1.709 |
| CP | 3.37 | 1.344 |
| ICP | 3.28 | 0.751 |
| CP | 4.43 | 3.442 |
| ICP | 1.57 | 1.062 |
| CP | 5.24 | 4.982 |
### Chart: UGT
| Category | RNA-seq | qPCR |
|---|---|---|
| ICP | 3.36 | 1.332 |
| CP | 4.53 | 1.551 |
| ICP | 3.55 | 14.403 |
| CP | 7.33 | 21.606 |
| ICP | 1.38 | 1.432 |
| CP | 5.0 | 5.321 |*
*
*
*
*
*
*
*
*
*
*
*
*
*
*
*
*
*
*
*
*
*
*
*
*
*
### Chart: ME
| Category | RNA-seq | qPCR |
|---|---|---|
| ICP | 4.25 | 0.931 |
| CP | 8.92 | 1.597 |
| ICP | 0.38 | 1.95 |
| CP | 0.08 | 0.882 |
| ICP | 4.7 | 0.94 |
| CP | 8.36 | 2.289 |
### Chart: PR1
| Category | RNA-seq | qPCR |
|---|---|---|
| ICP | 2.62 | 1.147 |
| CP | 2.42 | 1.682 |
| ICP | 3.38 | 3.474 |
| CP | 6.09 | 17.692 |
| ICP | 1.15 | 1.819 |
| CP | 2.68 | 4.834 |
### Chart: DXS
| Category | RNA-seq | qPCR |
|---|---|---|
| ICP | 3.22 | 1.593 |
| CP | 3.6 | 1.326 |
| ICP | 3.39 | 4.218 |
| CP | 6.19 | 7.899 |
| ICP | 1.07 | 0.54 |
| CP | 2.1 | 1.853 |*
*
*
*
*
*
*
*
*
*
*
*
*
*
*
*
*
*
*
*
*
*
*
Fig. S1. Comparison between relative expression levels determined by RNA-seq and RT-qPCR analyses. The sequences of primers used in RT-qPCR analysis (including primers for reference gene) are listed in Table S3. The asterisk (*) indicate statistically significant difference with p < 0.05 .
